# Supplementary material for: Evidence that endosperm turgor pressure both promotes and restricts seed growth and size
Source: Nat Commun. 2023 Jan 5;14:67. doi: 10.1038/s41467-022-35542-5 (PMC9814827; doi:10.1038/s41467-022-35542-5)
Supplement: Supplementary file 1 — Supplementary Information [file 41467_2022_35542_MOESM1_ESM.pdf]

Evidence that endosperm turgor pressure both promotes and restricts seed growth and size

Audrey Creff, Olivier Ali, Camille Bied,
Vincent Bayle, Gwyneth Ingram, Benoit Landrein.

Supplementary Information:

Supplementary Figures

Supplementary Tables

Supplementary Note

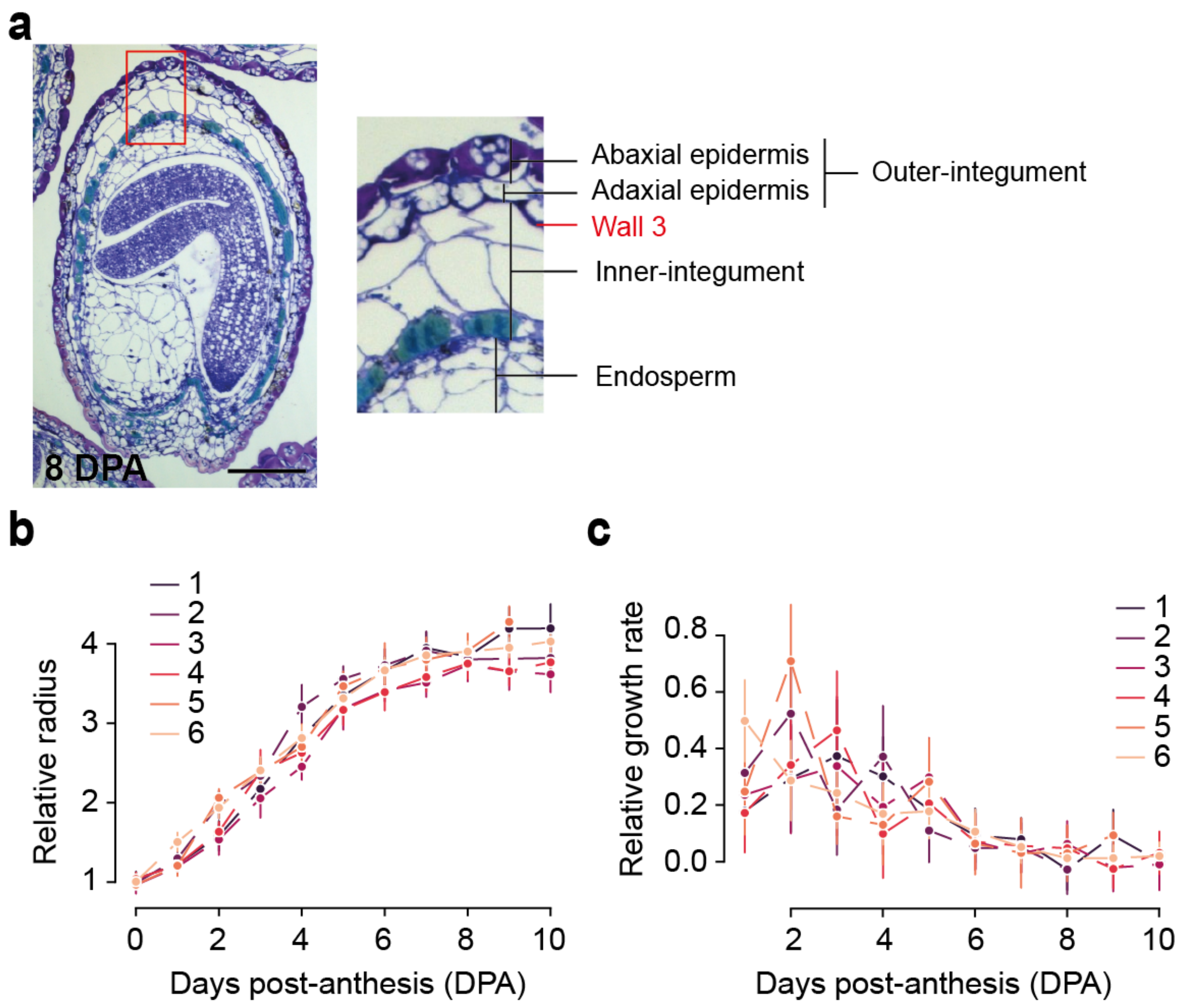

Supplementary Figure 1. **Structural organization and growth pattern of WT seeds**
a Col-0 seed at 8 DPA (Days post anthesis) stained with toluidine blue. The close-up view shows the organization of the testa layers and the presence of wall 3 separating the inner and outer-integuments. Scale bar: 100 µm. **b** and **c** Mean WT seed area (**b**) and relative growth rate (**c**) as a function of time (in DPA) in 6 independent experiments. The dots show the mean and the error bars show the standard deviation. Replicate 1: 0DPA: n=32, 1DPA: n=62, 2DPA: n=73, 3DPA: n=66, 4DPA: n=64, 5DPA: n=57, 6DPA, n=44, 7DPA: n=55, 8DPA: n=33, 9DPA: n=59, 10DPA: n=50; replicate 2: 0DPA: n=61, 1DPA: n=53, 2DPA: n=65, 3DPA: n=57, 4DPA: n=78, 5DPA: n=63, 6DPA, n=80, 7DPA: n=80, 8DPA: n=92, 9DPA: n=0, 10DPA: n=109; replicate 3: 0DPA: n=74, 1DPA: n=105, 2DPA: n=109, 3DPA: n=103, 4DPA: n=105, 5DPA: n=94, 6DPA, n=120, 7DPA: n=67, 8DPA: n=103, 9DPA: n=103, 10DPA: n=96; replicate 4: 0DPA: n=110, 1DPA: n=75, 2DPA: n=100, 3DPA: n=103, 4DPA: n=94, 5DPA: n=102, 6DPA, n=85, 7DPA: n=98, 8DPA: n=104, 9DPA: n=104, 10DPA: n=108; replicate 5: 0DPA: n=57, 1DPA: n=90, 2DPA: n=83, 3DPA: n=57, 4DPA: n=69, 5DPA: n=89, 6DPA, n=87, 7DPA: n=90, 8DPA: n=69, 9DPA: n=91, 10DPA: n=0, Replicate 6: 0DPA: n=94, 1DPA: n=100, 2DPA: n=99, 3DPA: n=97, 4DPA: n=100, 5DPA: n=106, 6DPA, n=89, 7DPA: n=103, 8DPA: n=100, 9DPA: n=89, 10DPA: n=84.

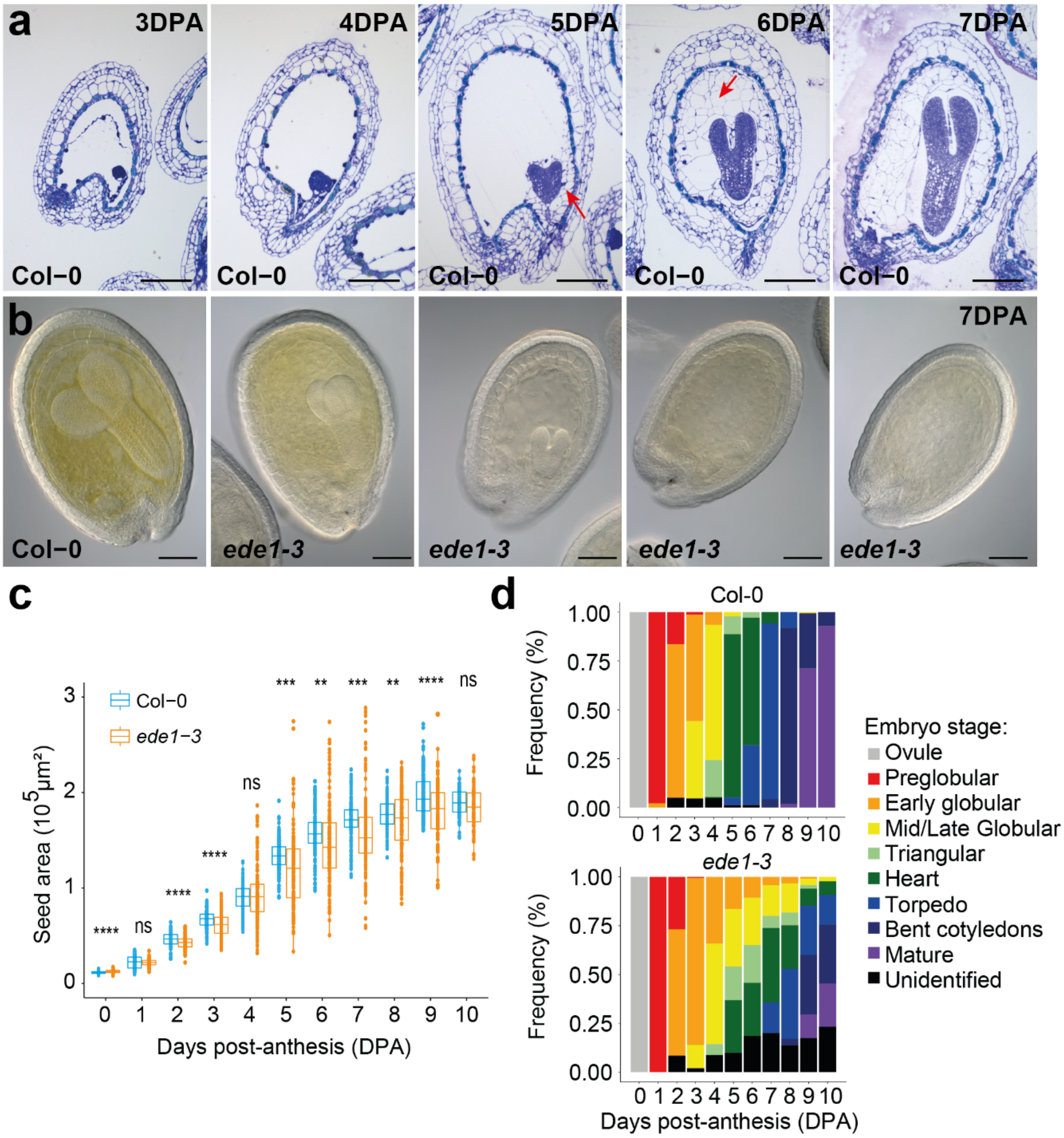

Supplementary Figure 2. **Endosperm cellularization does not strongly affect seed growth**
a WT seeds stained with toluidine blue at different stages of development (DPA: days post-anthesis). The red arrows mark the initiation and progression of cellularization from 5 DPA onwards. Scale bars: 100 µm. **b** WT and *ede1-3* at 7 DPA showing embryo growth defects in the mutant. Scale bars: 100 µm. **c** Seed area as a function of time in Col-0 and *ede1-3 (*two independent experiments; Col-0: 0DPA: n=151, 1DPA: n=190, 2DPA: n=182, 3DPA: n=154, 4DPA: n=169, 5DPA: n=195, 6DPA, n=176, 7DPA: n=193, 8DPA: n=169, 9DPA: n=180, 10DPA: n=84; *ede1-3*: 0DPA: n=153, 1DPA: n=187, 2DPA: n=119, 3DPA: n=158, 4DPA: n=161, 5DPA: n=152, 6DPA: n=140, 7DPA: n=160, 8DPA: n=153, 9DPA: n=115, 10DPA: n=86). The centerline shows the median; the box limits show the upper and lower quartiles, the whiskers correspond to 1.5x interquartile range. Single points are superimposed on the boxplots. Data were compared using two-sided Student tests without adjustments for multiple comparisons, ** $p<0.01$, *** $p<0.001$, **** $p<0.0001$. **d** Classification of the embryos in the batches of Col-0 and *ede1-3* seeds presented in (c) (two independent experiments; Col-0: 0DPA: n=151, 1DPA: n=190, 2DPA: n=182, 3DPA: n=154, 4DPA: n=169, 5DPA: n=195, 6DPA, n=176, 7DPA: n=193, 8DPA: n=169, 9DPA: n=180, 10DPA: n=84; *ede1-3*: 0DPA: n=153, 1DPA: n=187, 2DPA: n=119, 3DPA: n=158, 4DPA: n=161, 5DPA: n=152, 6DPA: n=140, 7DPA: n=160, 8DPA: n=153, 9DPA: n=115, 10DPA: n=86). The “unidentified” class corresponds to seeds where the embryo was not visible or, in the case of *ede1-3* at late stages of development, displayed defects similar to some presented in panel (b) that precluded classification.

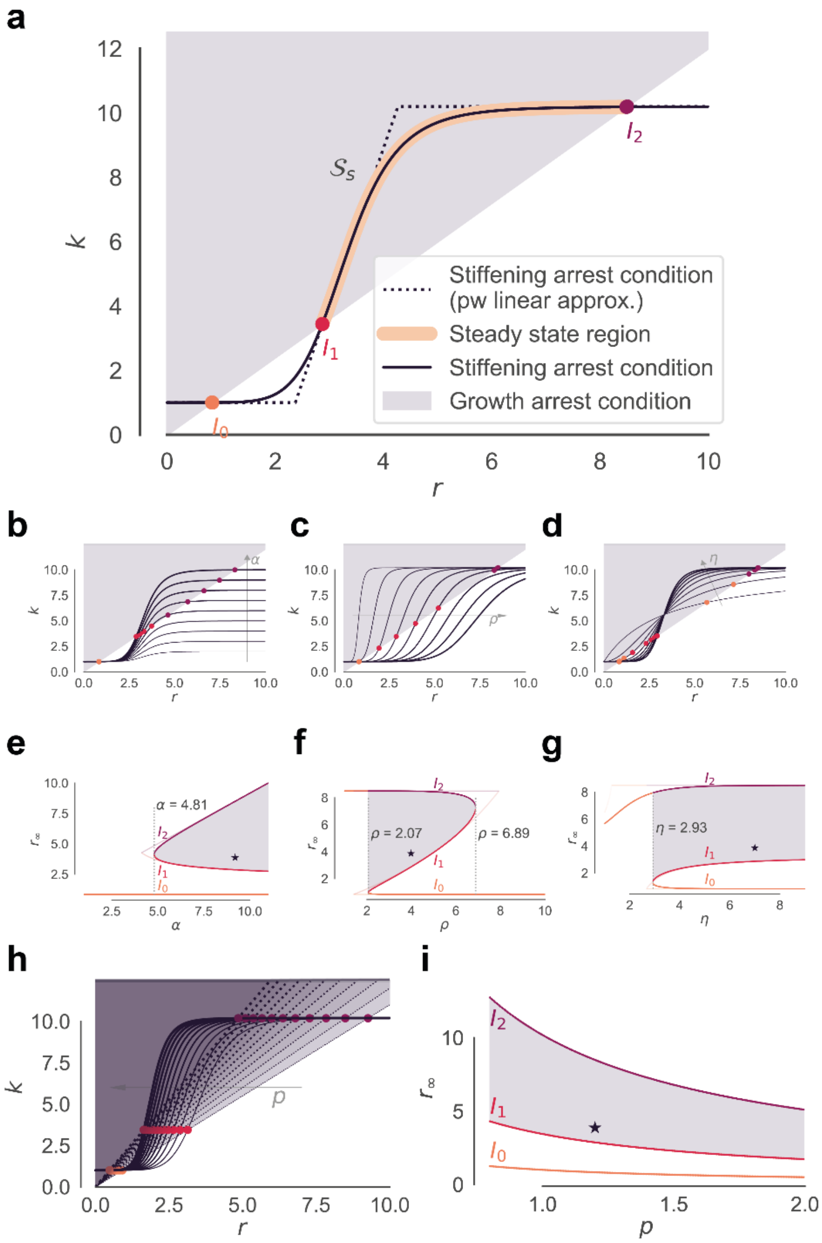

Supplementary Figure 3. **Theoretical analysis of the model steady state solutions**
a Visualization of the steady state (S_s) within the configuration space (plane *{r,k}*). The continuous black curve corresponds to the stiffening arrest condition, *i.e.* vanishing of equation (7) first line (Supplementary Note). The intersection of both gives the steady state, in yellow. The dashed curve corresponds to the asymptotic case where the Hill function in the stiffening equation is replaced by its piecewise linear approximation given in equation (10). Points I_0, I_1 and I_2 indicate roots of equation (8) and correspond to the boundaries of the steady state region. The values of the system parameters used to plot this figure correspond to those of the simulation best-fitting Col-0 experimental data and given in Supplementary Table 4. **b**,**c**,**d** Evolution of the steady state as parameters α, ρ and η vary. **e**, **f**, **g** Evolution of the roots of equation (3), corresponding to the abscissae of the boundary points I_0, I_1 and I_2 within the configuration space as functions of the model parameters. The gray area corresponds to zones where steady state solutions of equation (7) exist. The star in each panel corresponds to the result of the simulation best fitting Col-0 experimental data (Supplementary Table 4). The shadowed curves correspond to the piecewise linear approximated problem given in equation (10). Note that each panel (e,f,g) depicts the same evolution as that directly above, resp. (b, c, d). **h** Evolution of the steady state region within the configuration space as the endosperm pressure increases. **i** Evolution of the roots of equation (3) as functions of the endosperm pressure value. The star depicts the position of the simulation best-fitting *Col-0* experimental data.

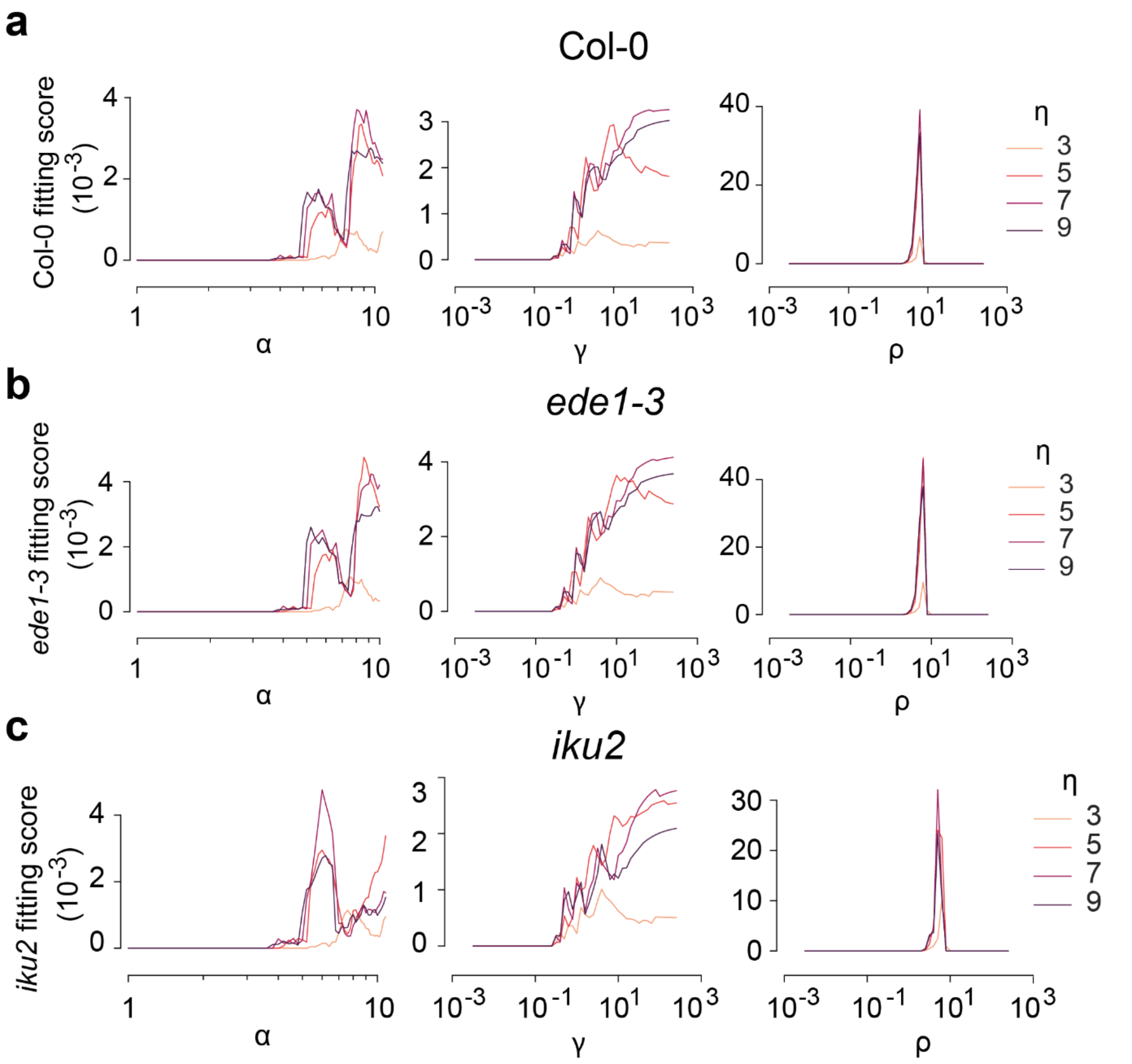

Supplementary Figure 4. **Fitting of the simulations to the experimental data**
Fitting score of the simulations using a constant pressure as input to the experimental measurements of seed size in Col-0 (a), *ede1-3* (b) and *iku2* (c) as a function of four parameters characterizing stress-dependent shell stiffening: (α) amplitude of stiffening, (γ) threshold ratio between stiffening and growth, (ρ) steepness of the stiffening mechanism (Hill function exponent), (η) characteristic time ratio between growth and stiffening.

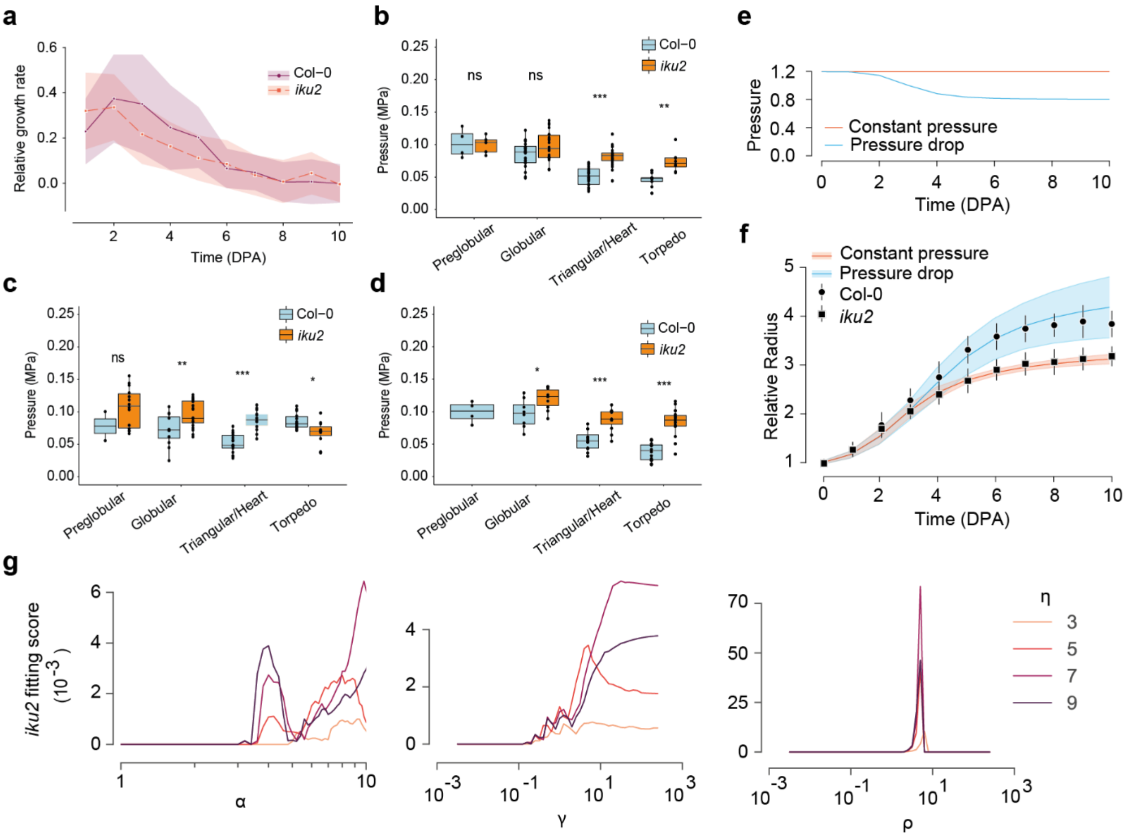

Supplementary Figure 5. **Characterization of *iku2* defects in endosperm pressure and seed growth**

a Relative Col-0 and *iku2* seed growth rate obtained by deriving the seed size measurements presented in Fig.2c. The thick curves correspond to the mean behavior and the shadowy bands to the calculated deviation (Three independent experiments, Col-0: 0DPA: n=216, 1DPA: n=242, 2DPA: n=282, 3DPA: n=272, 4DPA: n=263, 5DPA: n=253, 6DPA: n=249, 7DPA: n=220, 8DPA: n=240, 9DPA: n=266, 10DPA: n=254; *iku2*: 0DPA: n=195, 1DPA: n=241, 2DPA: n=289, 3DPA: n=262, 4DPA: n=271, 5DPA: n=268, 6DPA: n=268, 7DPA: n=247, 8DPA: n=237, 9DPA: n=237, 10DPA: n=268). **b-d** Endosperm pressure in Col-0 and *iku2* seeds extracted from stiffness measurements measured using a nanoindenter in two experiments independent from that presented in Fig. 2d. Seeds were classified based on the developmental stage of their embryo (b: Col-0: preglobular: n=10, globular: n=66, triangular/heart: n=37, torpedo: n=19; *iku2*: preglobular: n=21, globular: n=8, triangular/heart: n=14, torpedo: n=54, c: Col-0: preglobular: n=2, globular: n=12, triangular/heart: n=21, torpedo: n=14; *iku2*: preglobular: n=14, globular: n=20, triangular/heart: n=14, torpedo: n=11, d: Col-0: preglobular: n=4, globular: n=8, triangular/heart: n=12, torpedo: n=16; *iku2*: globular: n=10, triangular/heart: n=11, torpedo: n=19). The centerline shows the median; the box limits show the upper and lower quartiles, the whiskers correspond to 1.5x interquartile range. Single points are superimposed on the boxplots. Data were compared using two-sided Student tests without adjustments for multiple comparisons, * $p<0.05$ ** $p<0.01$, *** $p<0.001$. **e** Shape of the pressure drop implemented in the simulations to reproduce the changes in pressure measured experimentally in WT seeds in panel (d). **f** Relative seed radius as a function of time in Col-0 and *iku2* in experiments and simulations. The black circles and squares with bars represent the mean and SD of the experimental measurements of Col-0 and *iku2* seed size. The solid lines and shadowed bands show the mean and the standard deviation of the 100 best simulations where the model was fitted to *iku2* at a constant pressure of 1.2 or where the pressure drop shown in e was implemented. **g** Fitting score of the simulations to the experimental measurements of *iku2* seed size obtained using a time-dependent function (as shown in Fig.2e) to quantitatively fit the average pressure drop measured experimentally in *iku2* as input for pressure as a function of four parameters characterizing stress-dependent shell stiffening: (α) amplitude of stiffening, (γ) threshold ratio between stiffening and growth, (ρ) steepness of the stiffening mechanism (Hill function exponent), (η) characteristic time ratio between growth and stiffening.

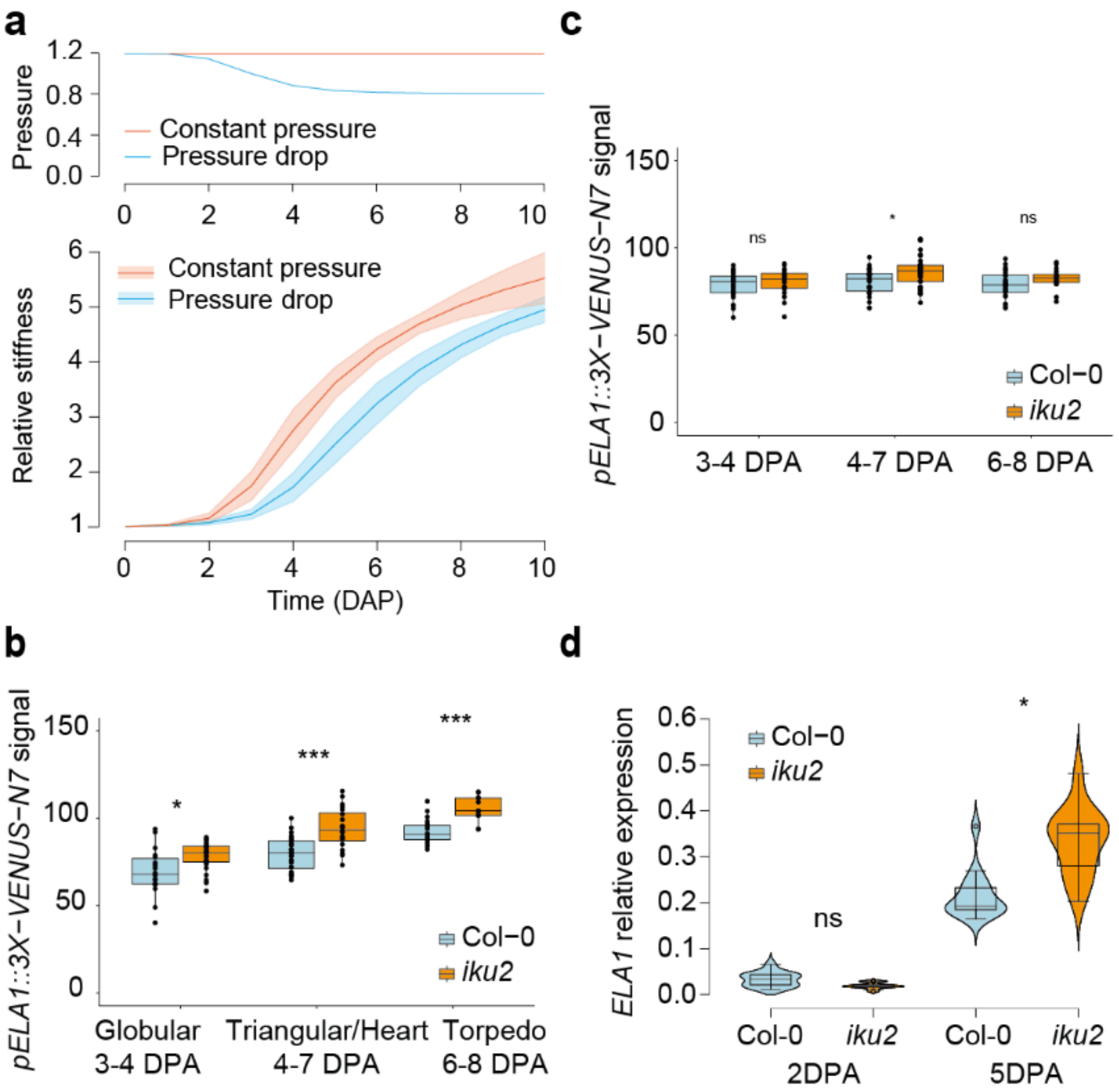

Supplementary Figure 6. **Reducing pressure delays mechanosensitive testa stiffening**
a Relative testa stiffness as a function of time in the 100 best simulations after model fitting to *iku2* data at a constant pressure of 1.2 or with a pressure drop implemented. Thick curves and shadowed bands correspond to the mean behavior and standard deviation respectively. **b,c** Mean signal of the *pELA1::3X-VENUS-N7* reporter in nuclei of Col-0 and *iku2* seeds (intensity unit/pixel) in two experiments independent from the one presented in Fig.3c. Seeds were classified according to the developmental stage of their embryo stage (b. Col-0, Globular: n=18, Triangular/Heart: n=28, Torpedo: n=23; *iku2*, Globular: n=35, Triangular/Heart: n=20, Torpedo: n=12; c. Col-0, Globular: n=26, Triangular/Heart: n=26, Torpedo: n=25; *iku2*, Globular: n=25, Triangular/Heart: n=34, Torpedo: n=20). The centerline shows the median; the box limits show the upper and lower quartiles, the whiskers correspond to 1.5x interquartile range. Single points are superimposed on the boxplots. Data were compared using two-sided Student tests without adjustments for multiple comparisons, * p<0.05, ** p<0.01, *** p<0.001. **d** Relative *ELA1* expression in WT and *iku2* seeds at 2 and 5 DPA assessed by qPCR. Pool of three independent experiments of respectively 5, 4 and 5 biological replicates. Superimposed on the violin plots, in the boxplots, the centerline shows the median; the box limits show the upper and lower quartiles, the whiskers correspond to 1.5x interquartile range and single points show outliers. Data were compared using a Kruskall-Wallis test followed by a Dunn multiple comparison post-hoc test, * p<0.05.

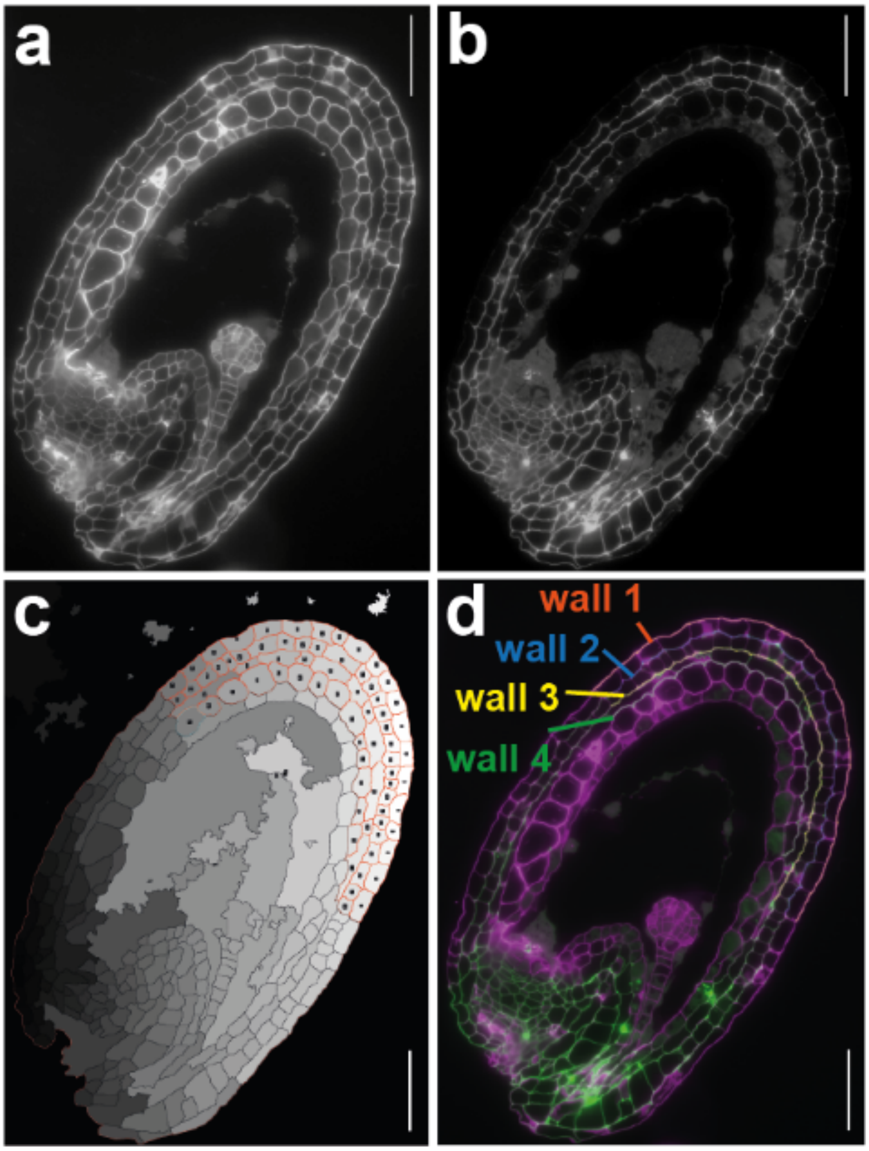

Supplementary Figure 7 **Quantification of immunofluorescence signal in testa walls following immunostaining of cell wall components**

a Control channel (stained with calcofluor). **b** Signal channel (stained with the LM19 antibody). **c** Segmentation of testa cells using ImageJ and layer assignment. **d** Extraction and classification of the periclinal walls of the testa. Final overlay shows the cell wall ROI (Regions Of Interest) used for the quantification. Scale bars: 50 µm.

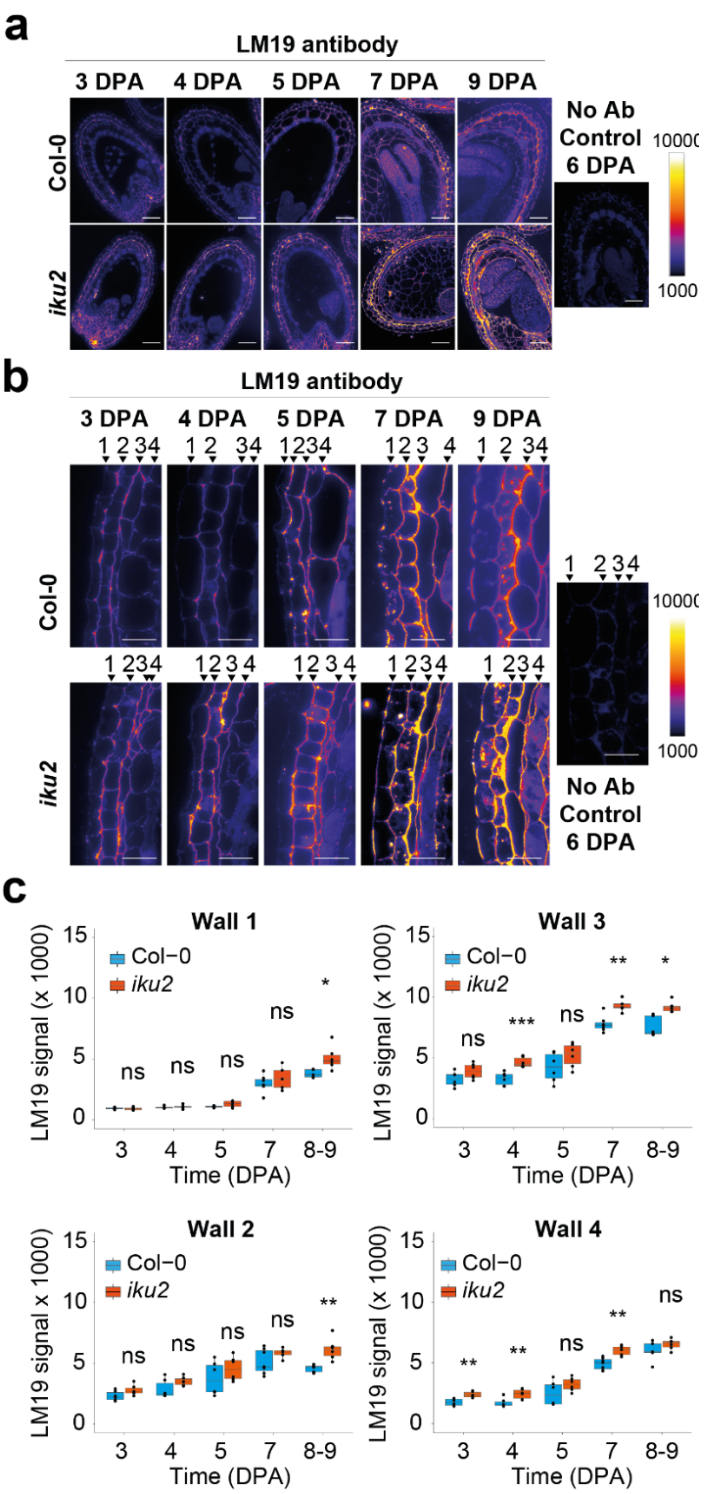

Supplementary Figure 8. **LM19 signal in testa walls**

a,b Signal of the LM19 antibody in Col-0 and *iku2* testa walls obtained by immunolocalization of seed sections at different stages of development (intensities are color-coded using the fire lookup table). Wall numbers (1 to 4) are displayed in the close-up views (b). Scale bars: a, 50 µm and b, 20µm. **c** Signal intensity (x 10^3 intensity unit/pixel) of the LM19 antibody in periclinal testa walls 1 to 4 (counting from the outside of the seed) of Col-0 and *iku2* seeds as a function of time (two independent experiments, Col-0: 3DPA: n=9, 4DPA: n=9, 5DPA: n=10, 6DPA: n=8, 7-9DPA: n=9; *iku2*: 3DPA: n=9, 4DPA: n=9, 5DPA: n=9, 6DPA: n=9, 7-9DPA: n=9). The centerline shows the median; the box limits show the upper and lower quartiles, the whiskers correspond to 1.5x interquartile range. Single points are superimposed on the boxplots. Data were compared using two-sided Student tests without adjustments for multiple comparisons, * p<0.05, ** p<0.01, *** p<0.001.

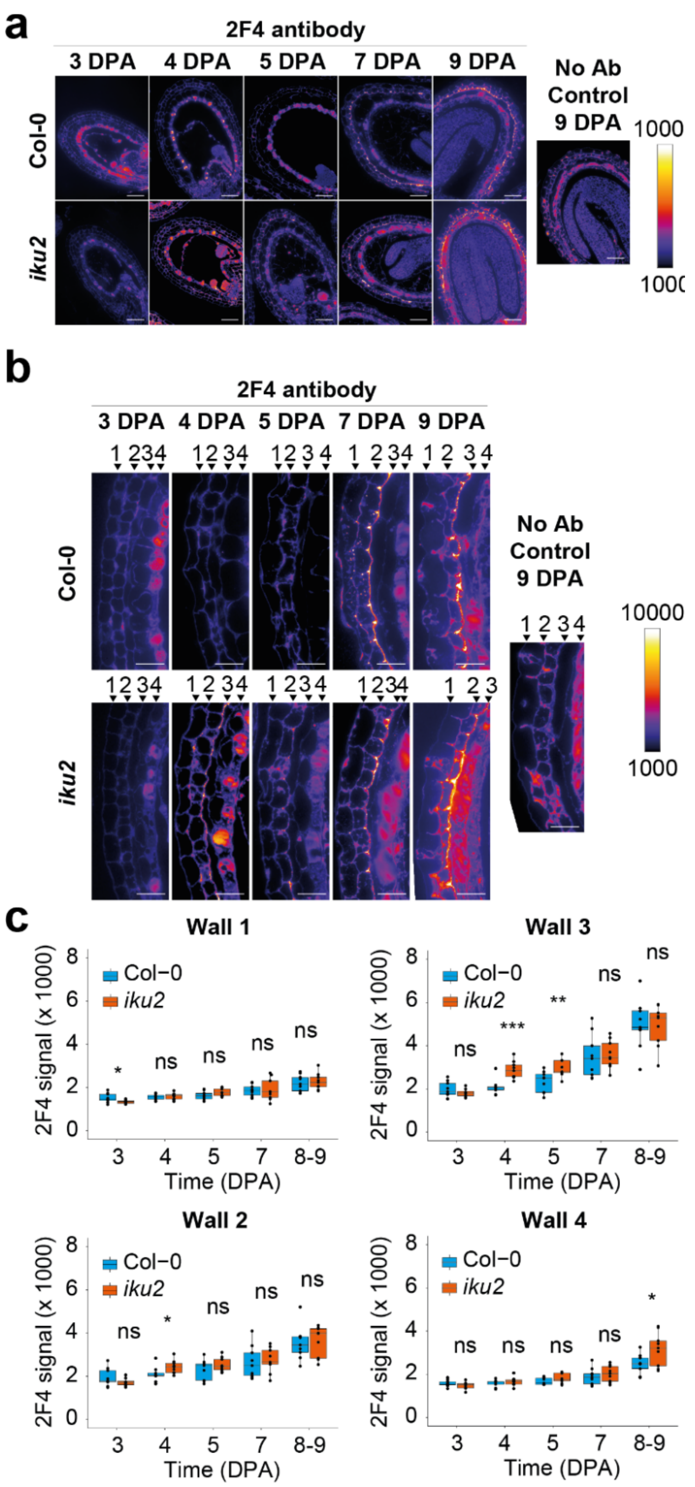

Supplementary Figure 9. **2F4 signal in testa walls**

a,b Signal of the 2F4 antibody in Col-0 and *iku2* testa walls obtained by immunolocalization of seed sections at different stages of development (intensities are color-coded using the fire lookup table). Wall numbers (1 to 4) are displayed in the close-up views (a). Scale bars: a, 50 µm and b, 20µm. **c** Signal intensity (x 10^3 intensity unit/pixel) of the 2F4 antibody in periclinal testa walls 1 to 4 (counting from the outside of the seed) of Col-0 and *iku2* seeds as a function of time (three independent experiments, Col-0: 3DPA: n=9, 4DPA: n=9, 5DPA: n=9, 6DPA: n=9, 7-9DPA: n=9; *iku2*: 3DPA: n=8, 4DPA: n=9, 5DPA: n=9, 6DPA: n=9, 7-9DPA: n=9). The centerline shows the median; the box limits show the upper and lower quartiles, the whiskers correspond to 1.5x interquartile range. Single points are superimposed on the boxplots. Data were compared using two-sided Student tests without adjustments for multiple comparisons, * p<0.05, ** p<0.01, *** p<0.001.

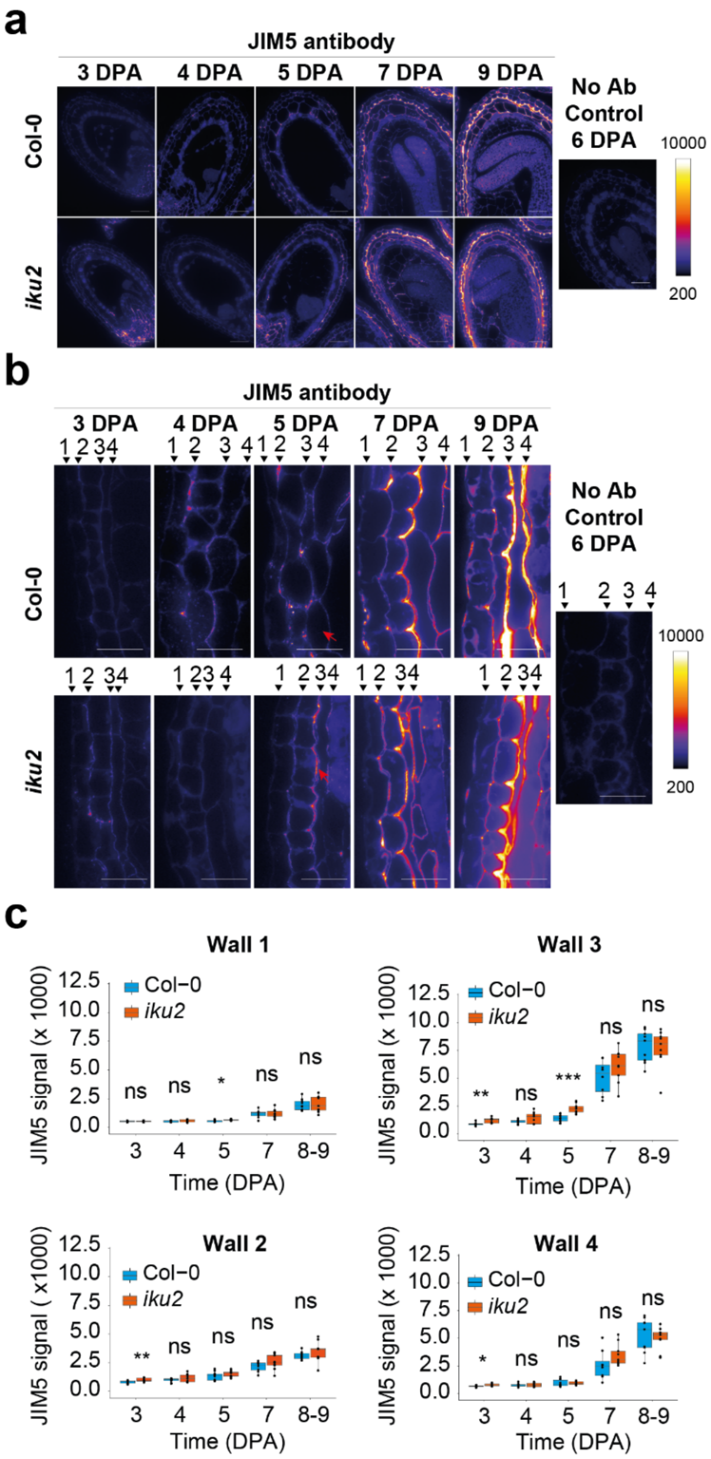

Supplementary Figure 10. **JIM5 signal in testa walls**

a,b Signal of the JIM5 antibody in Col-0 and *iku2* testa walls obtained by immunolocalization of seed sections at different stages of development (intensities are color-coded using the fire lookup table). Wall numbers (1 to 4) are displayed in the close-up views (b). Scale bars: a, 50 µm and b, 20µm. **c** Signal intensity (x 10^3 intensity unit/pixel) of the JIM5 antibody in periclinal testa walls 1 to 4 (counting from the outside of the seed) of Col-0 and *iku2* seeds as a function of time (pool of three independent experiments, Col-0: 3DPA: n=9, 4DPA: n=9, 5DPA: n=10, 6DPA: n=8, 7-9DPA: n=9; *iku2*: 3DPA: n=9, 4DPA: n=9, 5DPA: n=9, 6DPA: n=9, 7-9DPA: n=9). The centerline shows the median; the box limits show the upper and lower quartiles, the whiskers correspond to 1.5x interquartile range. Single points are superimposed on the boxplots. Data were compared using two-sided Student tests without adjustments for multiple comparisons, * $p<0.05$, ** $p<0.01$, *** $p<0.001$.

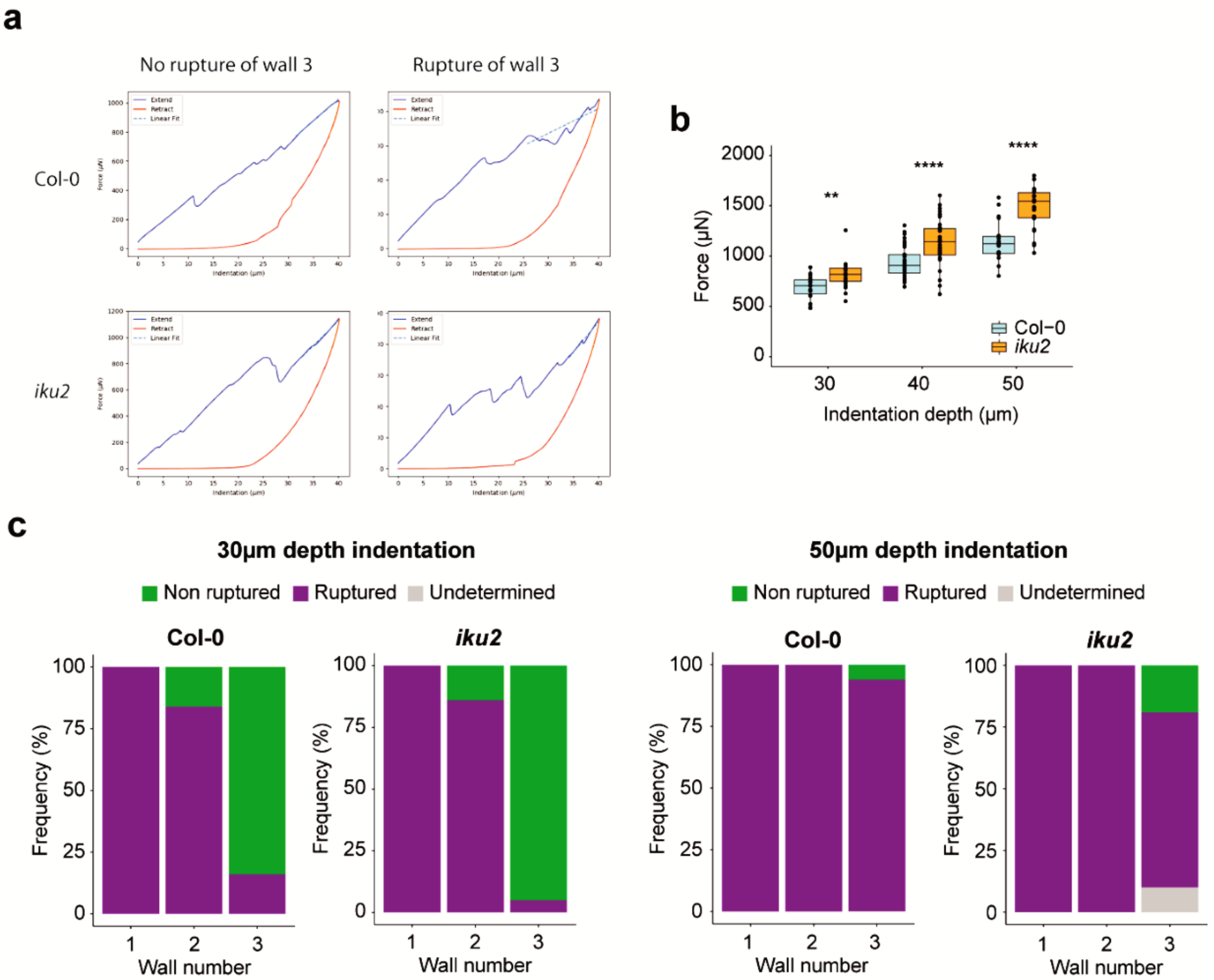

Supplementary Figure 11. **Measurements of testa wall rupture after indentation**
a Representative force/displacement curves obtained while performing a 40 µm indentation of Col-0 and *iku2* seeds. **b** Force needed to perform 30 µm, 40 µm and 50 µm indentations and rupture testa walls in Col-0 and *iku2* (Two independent experiments, Col-0: 30µm depth: n=18, 40µm depth: n=34, 50µm depth: n=18, *iku2*: 30µm depth: n=24, 40µm depth: n=36, 50µm depth: n=22). The centerline shows the median; the box limits show the upper and lower quartiles, the whiskers correspond to 1.5x interquartile range. Single points are superimposed on the boxplots. Data were compared using two-sided Student tests without adjustments for multiple comparisons, ** p<0.01, *** p<0.001. **c** Quantification of testa wall rupture in Col-0 and *iku2* following a 30 µm or a 50 µm indentation with a conical tip (two independent experiments, 30µm indentation: Col-0: n=19, *iku2*: n=21; 50µm indentation: Col-0: n=18, *iku2*: n=21).

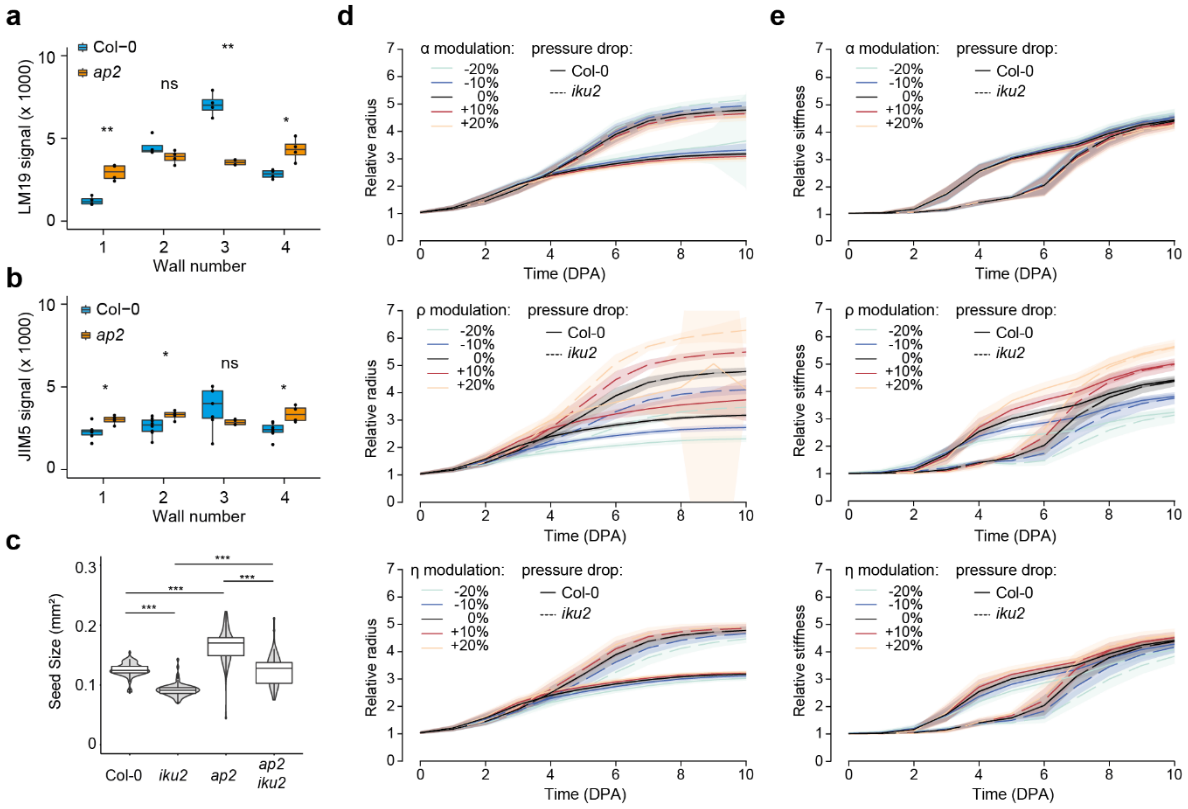

Supplementary Figure 12. ***iku2* seed size phenotype can be rescued by *ap2***

a,b Quantification of the signal on the LM19 (a) and JIM5 (b) antibodies in the 4 outermost testa walls of Col-0 and *ap2* seeds (one experiment independent from the one presented in Fig.4, LM19: Col-0: 4 seeds, *ap2-6*: 4 seeds; JIM5: Col-0: 7 seeds, *ap2-6*: 4 seeds). The centerline shows the median; the box limits show the upper and lower quartiles; the whiskers correspond to 1.5x interquartile range. Single points are superimposed on the boxplots. Data were compared using two-sided Student tests without adjustments for multiple comparisons, * $p<0.05$, ** $p<0.01$, *** $p<0.001$.. **c** Measurements of seed area in Col-0, *iku2*, *ap2-6* and *iku2 ap2-6* (one independent experiment: Col-0, n=61; iku2, n=79; ap2-6, n=69, ap2-6 iku2, n=60). Superimposed on the violin plots, in the boxplots, the centerline shows the median; the box limits show the upper and lower quartiles, the whiskers correspond to 1.5x interquartile range and single points show outliers. Data were compared using two-sided Student tests without adjustments for multiple comparisons, * $p<0.05$, ** $p<0.01$, *** $p<0.001$.. **d,e** Evolution of the relative radius (d) and of the relative stiffness (e) as a function of the stiffening parameter values (α, ρ, η) using either Col-0 or iku2 drop functions as input for pressure. All simulations were performed on the 100 simulations best-fitting *iku2* seed size using *iku2* drop of pressure as an input parameter (as shown in Fig.2e). Thick curves (plain for Col-0 and dotted for *iku2*) and shadowed bands correspond to the mean behavior and standard deviation respectively.

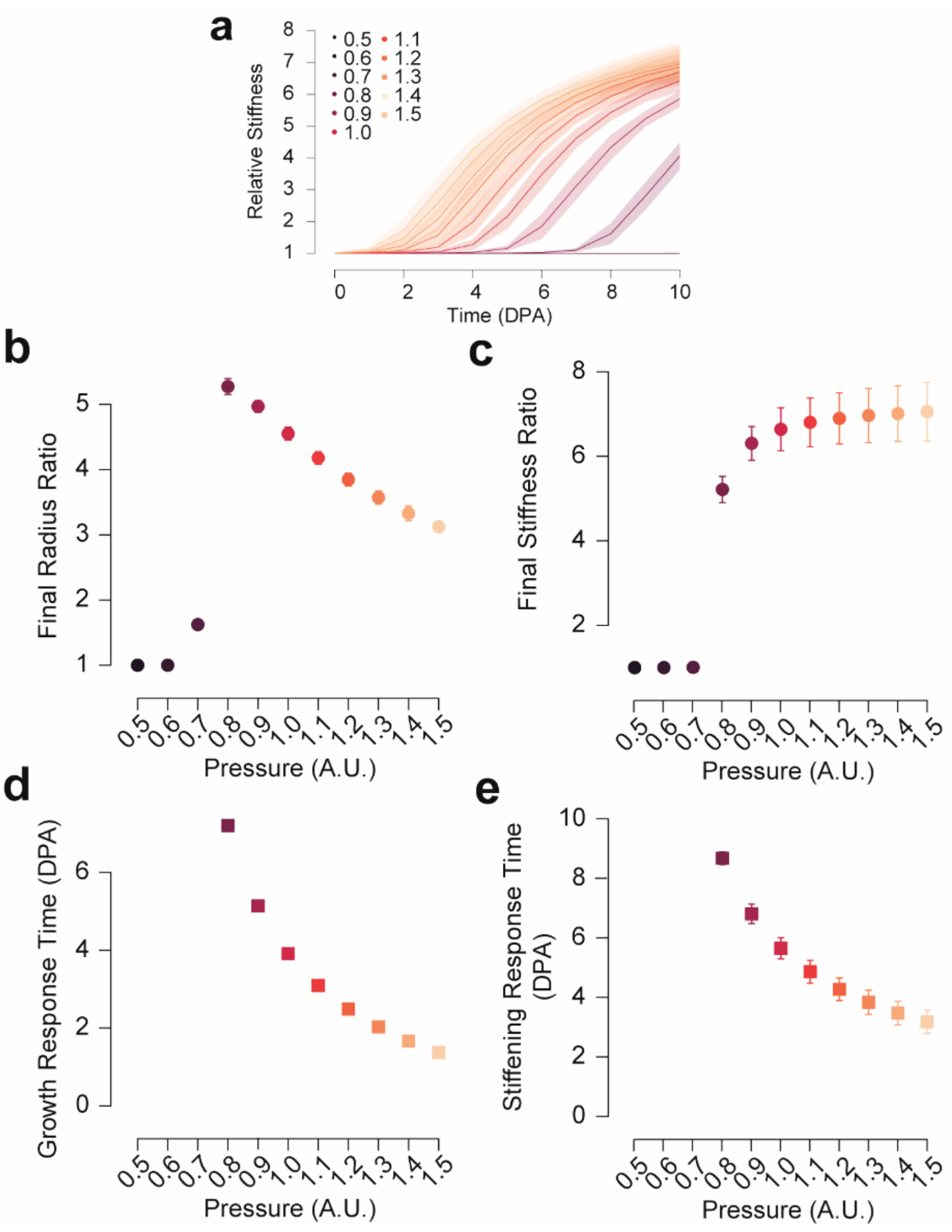

Supplementary Figure 13. **Effect of pressure modulations on growth and stiffening in simulations**

a Relative stiffness as a function of time and pressure in simulations. The thick curves and shadowed bands correspond to mean behavior and standard deviation of the 100 simulations best fitting the WT experimental data. **b-e** Evolution of radius and stiffness steady state and response time of the expanding shell when loaded with increasing values of pressure. (b and c): Ratio between the final and initial values of the relative radius (b) and stiffness (c) for increasing values of pressure. (d and e): Response time (defined as the time needed to reach half of the steady state value) of the growth process (d) and the stiffening process (e) for increasing values of pressure. Each point corresponds to 100 simulations, performed with the same constant value of pressure. Error bars depict the standard deviation. In d and e, the first three points of each graph, corresponding to pressure values of 0.5 to 0.7 are missing, as the system does not evolve for these small values of pressure.

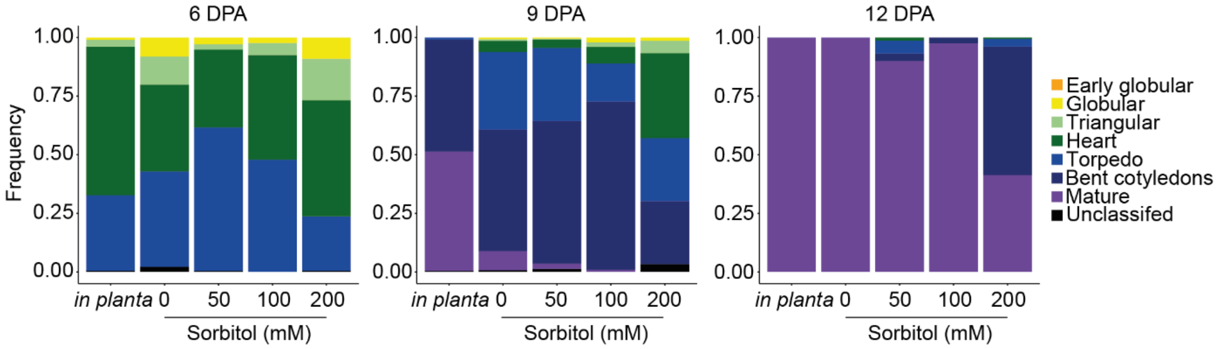

Supplementary Figure 14. **Increasing sorbitol concentration in the culture medium delays seed growth in fruits cultivated *in vitro*.**

Classification of the embryos in the batches of Col-0 seeds from fruits growing *in planta* or growing *in vitro* in culture media with increasing concentration of sorbitol from 3DPA onward (pool of two independent experiments; 6DPA: Uncut, n=202; 0mM Sorbitol, n=184; 50mM Sorbitol, n=214; 100mM Sorbitol, n=213; 200mM Sorbitol, n=198, 9DPA: Uncut, n=220; 0mM Sorbitol, n=145; 50mM Sorbitol, n=227; 100mM Sorbitol, n=99; 200mM Sorbitol, n=149; 12DPA: Uncut, n=209; 0mM Sorbitol, n=211; 50mM Sorbitol, n=161; 100mM Sorbitol, n=162; 200mM Sorbitol, n=179).

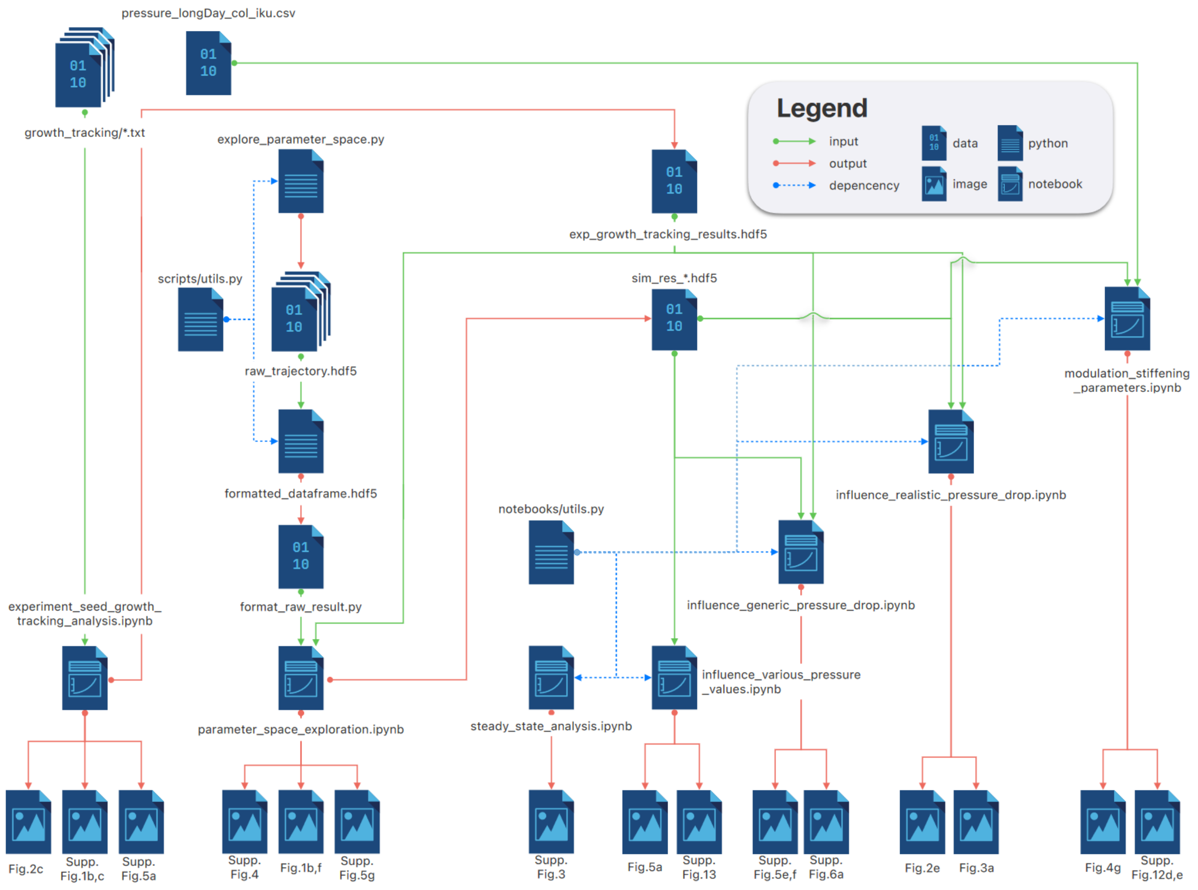

Supplementary Figure 15. **Workflow diagram of our simulation pipeline**

The experimental datasets (seed size and pressure measurements) used for simulations are shown on the top-right corner. The icons at the bottom depicts all the simulation-related figures provided within the main text and the supplementary materials. All python files (scripts, local libraries and notebooks) are available within the gitlab repository related to this manuscript and can be downloaded, run and modified. Detailed instructions on how to install and run this pipeline can be found within the gitlab repository. The original datasets can be found in the dedicated Zenodo repository and in the Source data File (see Supplementary Information).

Supplementary Tables

Param. & Var.	Meaning	Units	Values<br>*Ref.*
Geometry			
R	Radius of the spherical shell	μm	$47.5 - 310$ (measures)
H	Thickness of the shell	μm	$20 - 25$ (measures)
Mechanics			
P	Pressure exerted by the endosperm on the seed coat	MPa	$(3 - 18) \cdot 10^{-2}$ (measures)
K	Young's modulus of the shell	MPa	N/A
Growth			
τ_{g}	Growth characteristic time	s	N/A
$\varepsilon_{\mathrm{th}}$	Growth threshold (strain)	$\%$	N/A
Stiffening			
τ_{s}	Cell wall stiffening characteristic time	s	N/A
σ_{th}	Stiffening threshold (stress)	MPa	N/A
k^{0}_{on}	Intrinsic stiffening rate	$\mathrm{MPa} \cdot \mathrm{s}^{-1}$	N/A
$\Delta k^{\sigma}_{\mathrm{on}}$	mechanosensitive increase of the stiffening rate	$\mathrm{MPa} \cdot \mathrm{s}^{-1}$	N/A
η	Hill function exponent, steepness of the mechanosensitive stiffening mechanics	$\emptyset$	N/A
K_0	Stationnary value of the bulk rigidity modulus without mechanosensitive stiffening at play.	MPa	N/A

Supplementary table 1: **Variables & parameters.**

	Name	Symbol	Expression	Interpretation	Value/Range
**Variables**	Relative time	$\tilde{t}$	t/τ_{g}	Time normalized by the growth characteristic time.	$[0, 3]$
	Relative radius	r	R/H	Radius of the seed normalized by the thickness of the seed coat.	1.5
	Relative stiffness	k	K/K_0	Seed coat effective stiffness normalized by its stationary value when stress-induced process is active.	1
	Relative pressure	p	$P/(2\varepsilon_{\mathrm{th}}K_0)$	Endosperm pressure divided by the growth threshold times the basal value of the effective seed coat stiffness.	$[0.5, 2]$
**Parameters**	Stress-stiffening strength	α	$\Delta k_{\mathrm{on}}^{\sigma}/k_{\mathrm{on}}^{0}$	Maximum relative stiffness increase due to the stress-induced mechanism.	$[1, 11]$
	Characteristic time ratio	γ	$\tau_{\mathrm{g}}/\tau_{\mathrm{s}}$	Ratio between growth and stiffening characteristic times.	$[10^{-2,5}, 10^{2,5}]$
	Threshold ratio	ρ	$\sigma_{\mathrm{th}}/(\varepsilon_{\mathrm{th}}K_0)$	Ratio between stiffening and growth thresholds.	$[10^{-2,5}, 10^{2,5}]$
	Hill exponent	η	—	Steepness of the mechanosensitive stiffening mechanics.	$\{3, 5, 7, 9\}$

Supplementary table 2: **Dimensionless variables & parameters used within the dimensionless system, see 7.** The given values and ranges correspond to the ones used and explored within the parameter space simulations.

	Reference value	Explored range	Increment
α	9.20	$[1, 9]$	10^{-2}
ρ	3.98	$[1, 11]$	10^{-2}
η	7.00	$[1, 9]$	10^{-2}
p	1.20	$[0.8, 2]$	$5 \cdot 10^{-3}$

Supplementary table 3: **Parameter values used during the numerical analysis of 9.** The reference values corresponds to the paramter values of the WT-best-fitting simulation, see 5, obtained during our parameter space exploration campaign.

Variable	Min. value	Max. Value	Increment	Samples	Scale
α	1	11	.2	50	linear
η	3	9	1	4	linear
γ	-2.5	2.5	.1	50	logarithmic
ρ	-2.5	2.5	.1	50	logarithmic

Supplementary table 4: **Details of the sampling used to perform the parameter space exploration.**

		WT (Col-0)	*iku2*	*ede1-3*
**Best fit**	**Score (absolute value)**	3.86	5.87	2.66
	**Simulation** Id	228781	53830	196381
**Parameters**	**values**			
α	best:	9.20	5.20	6.60
	mean:	8.73	6.85	8.56
	median:	8.80	6.40	8.60
	st. dev.:	0.88	1.50	0.82
η	best:	7	9	7
	mean:	6.30	6.48	6.28
	median:	7.00	7.00	7.00
	st. dev.:	1.14	1.12	1.11
γ	best:	1.00	1.26	1.58
	mean:	60.4	64.3	64.3
	median:	28.4	31.6	40.0
	st. dev.:	74.0	74.5	72.5
ρ	best:	3.98	3.16	3.98
	mean:	5.88	4.76	5.93
	median:	6.31	5.01	6.31
	st. dev.:	0.80	0.76	0.77

Supplementary table 5: **Results from the parameter space exploration campaign:** parameter values that correspond to simulations fitting experimental data the best. The mean, median and standard deviation (st. dev.) values have been computed over the 100 best fitting results.

		**Percentage of outliers** (%)	
		WT (Col-0)	*iku2*
**Parameters**	**Modulation** (%)		
α	-20	2	5
	-10	2	2
	0	6	0
	10	6	3
	20	5	5
ρ	-20	6	6
	-10	2	4
	0	6	0
	10	5	3
	20	6	4
η	-20	6	1
	-10	3	1
	0	6	0
	10	5	0
	20	5	0

Supplementary table 6: **Number of diverging trajectories during stiffening modulation simulations.** For each parameter and each modulation value, we performed 100 simulations. Among those, some yield unrealistic final radii. We report in this table the number of such ill simulations.

Primer	Sequence	Application
Prom-IKU2-B4	ggggacaactttgtatagaaaagttgGGTCTCTCTTGA TAACGATTTG	Cloning of pIKU2
Prom-IKU2-B1R	ggggactgctttttgtacaaacttgTGTTCTCTACGTCG GAAGG	Cloning of pIKU2
iku2-Del-For	TTGCTGGAGAAGCTTGTTCTAG	Genotyping of *iku2*
iku2-Del-Rev	GAACTCCATGGGAATA-TTCCAG	Genotyping of *iku2*
AP2-CDS-F	GGGACAAGTTTGTACAAAAAAGCAGGCTTA ATGTGGGATCTAAACGAC	Cloning of AP2-CDS and genotyping of *ap2* by sequencing
AP2-CDS-R	GGGGACCACTTTGTACAAGAAAGCTGGGTA TCAAGAAGGTCTCATGAG	Cloning of AP2-CDS and genotyping of *ap2* by sequencing
EIF4A-F	TTCGCTCTTCTCTTTGCTCTC	qPCR
EIF4A-R	GAACTCATCTTGTCCCTCAAGTA	qPCR
At5g46630-F	TCAGGTGCCAATGTTCACAGC	qPCR
At5g46630-R	ACCGCTCTTCTCCCAAACCTTG	qPCR
CYP714A1-F	TCAGCCTCAATGGCTTCACT	qPCR
CYP714A1-R	CGGTTTCCCATATCAAAGACTC	qPCR

Supplementary Table 7 **List of primers used in this study**

Supplementary Note

Contents

1 System formalization **2**
1.1 Geometrical description 2
1.2 Mechanical assumptions 2
1.3 Biological assumptions 2
1.4 Dimensionless formalization 4

2 Steady state analysis **5**
2.1 Rationale 5
2.2 Existence 5
2.2.1 Numerical estimations 5
2.2.2 Analytical study 6
2.3 Stability 7

3 Numerical simulations **8**
3.1 Parameter space exploration 9
3.2 Simulations with time dependent pressure 11
3.3 Modulation of the stiffening parameters 13

1 System formalization

1.1 Geometrical description

To investigate the antagonist effects of mechanical stress on seed development, we derived the leanest model possible. To that end, we assimilated the seed coat to a spherical shell of radius R and homogeneous thickness H, yielding a one-dimensional geometrical description of the seed (Fig. 1.c).

1.2 Mechanical assumptions

From a mechanical perspective, we assumed an homogeneous and isotropic elastic response of the shell to external loading. This enabled us to account for the shell overall elastic properties through a single parameter: its effective bulk rigidity modulus (K). Furthermore, assuming the linearity of this elastic response yielded the well known Hooke law, equation (2), relating strain (ε) and stress (σ) to the effective bulk rigidity modulus within the shell:

$$\sigma = K\varepsilon \tag{2}$$

The endosperm influence on the seed coat is limited to an hydrostatic pressure (P) applied to the shell from within.

We also considered growth to be a *quasi-static* phenomenon, *i.e.* a continuous succession of mechanical equilibria where the elastic response of the seed coat balances the pressure forces generated on it by the endosperm.

Given the spherical symmetry of our representation, this assumption yields the Laplace law, equation (3), relating tensile stresses, at mechanical equilibrium in the deformed configuration, within the seed coat to endosperm pressure and the geometrical properties of the shell:

$$\sigma = \frac{PR}{2H} \tag{3}$$

1.3 Biological assumptions

We considered two responses of the seed coat to the mechanical solicitation: Growth and cell wall stiffening.

Both mechanisms are highly complex processes involving numerous biomechanical and biochemical entities and mechanisms; *e.g.* production of cell wall components and modifications of their properties, cytoskeleton organization, gene expression, mechanosensitive response through transmembrane channels or receptor kinases. A formalization accounting exhaustively for all of the molecular processes at stake is of course out of reach and not in the scope of this work. To alleviate this complexity we opted for an *empirical* formalization of both phenomena.

Growth: We described growth within the seed coat with a thresholded strain-based law, equation (4).

$$\frac{1}{R}\frac{dR}{dt} = \frac{1}{\tau_{\text{g}}}\Big(\frac{\varepsilon}{\varepsilon_{\text{th}}} - 1\Big)_{+} \tag{4}$$

$$\text{with: } (x)_{+} = \begin{cases} x & \text{if } x \geq 0 \\ 0 & \text{else} \end{cases}$$

Equation (4) states that the relative growth rate of the seed coat is proportional to the strain above a given threshold (ε_{th}). If the strain remains below this threshold, the growth rate vanishes. The growth characteristic time (τ_{g}) quantifies the kinetics of this irresversible expansion. This empirical law can be seen as an extension of the original Lockhart (*1*) and Ortega (*2*) models. Such strain-based update of the seminal Lockhart equation has already been used in previous modeling works (*3–5*) to account for experimental observations, namely that cells expand orthogonally to the cell wall stiffest direction.

Cell wall stiffening: We formalized cell wall stiffening within the seed coat as a first-order ordinary differential equation, equation (5), expressing the stiffening rate of the cell wall as the combination of a *production* and a *degradation* term.

$$\frac{dK}{dt} = k_{\text{on}}^{0} + \Delta k_{\text{on}}^{\sigma} h_{\eta}\left(\sigma, \sigma_{\text{th}}\right) - \frac{K}{\tau_{\text{s}}} \tag{5}$$

The degradation term, third element of equation (5) *rhs*, is assumed to be linear and yields an exponential decay of characteristic time τ_{s} if no production term is considered.

The production term is composed by the first two elements of the right hand-side of equation (5). The first one depicts a *basal* stiffening rate (k_{on}^{0}). Combined with the degradation term, it provides the seed coat with a stationary value ($K_0 = k_{\text{on}}^{0}\tau_{\text{s}}$) for its effective bulk rigidity modulus; when no mechanobiological regulation is at play.

The second element of equation (5) *rhs* accounts for the seed coat stress-sensitive stiffening ability. It corresponds to a *Hill function*, equation (6), increasing the production rate from k_{on}^{0} to $k_{\text{on}}^{0} + \Delta k_{\text{on}}^{\sigma}$ when tensile stresses within the seed coat go from low values ($\sigma \ll \sigma_{\text{th}}$) to high ones ($\sigma \gg \sigma_{\text{th}}$).

$$h_{\eta}\left(\sigma, \sigma_{\text{th}}\right) = \frac{1}{1 + \left(\sigma/\sigma_{\text{th}}\right)^{-\eta}} \tag{6}$$

The parameter σ_{th} can be interpreted as a threshold for this stress-sensitive mechanism and the Hill exponent η as a measure of its non-linearity ($i.e.$ the bigger η the sharper the response to stress).

All parameters and variables used through equations (2) to (6) are listed in table (1).

1.4 Dimensionless formalization

To analyze the properties of the differential system composed by equations (4) and (5) we made it dimensionless by normalizing the three variables (t, R, K) by the three parameters ($\tau_{\rm g}$, H, K_0) respectively. This yield system (7).

$$\begin{cases} \dot{r} = \left(\frac{pr}{k} - 1\right)_+ r \\ \dot{k} = \gamma(1 - k + \alpha h_\eta\,(rp, \rho)) \end{cases} \tag{7}$$

N.B.: In system (7), derivation with respect to the dimensionless time variable ($\tilde{t}$) is noted with a dot over the derived quantity.

All the dimensionless parameters and variables of system (7) are listed within table (2).

Rationale: This dimensionless approach seems relevant in our case, for several reasons: It simplifies the system by removing intermediate variables (σ and ε) and it highlights the relationship between the structural properties of the equations and the dynamical properties of the system. But most importantly, since Equations (4) & (5) derive from empirical considerations, their parameters cannot be tracked to actual biochemical and/or rheological properties that could be properly measured experimentally. Parametrizing equations (4) & (5) with relevant values appears therefore difficult, if not impossible. By focusing on dimensionless versions of these equations, we alleviate this difficulty: The parameters we need to estimate correspond now to ratios between comparable quantities. The drawback of this approach is that we can only extract qualitative information from their analysis. For instance, the condition: $\gamma = 10 \Rightarrow \tau_{\rm g} = 10\tau_{\rm s}$, can only be translated into the following qualitative statement: *"growth is slower than the stiffening process by one order of magnitude."*

2 Steady state analysis

2.1 Rationale

Considering a dynamical system formalized by a set of Ordinary Differential Equations (*ODEs*), steady state solutions refer to specific values of the variables that cancel the time derivatives. They correspond to stationary configurations toward which the system might converge. Their study usually reveals important structural properties[1] of the system at stake. Steady state analysis often has a strong geometrical interpretation which helps grasping more intuitively these fundamental properties.

In the specific case we are interested in, system (7), such stationary configurations will be noted $\big(r_\infty, k_\infty\big)$. Beside the trivial solution $\big(r_\infty, k_\infty\big) = \big(0, 1\big)$, more interesting ones verify:

$$\begin{gathered} F(r_\infty) \geq 0 \\ \text{with:} \\ F: r \rightarrow 1 - rp + \alpha h_\eta\,(rp, \rho) \end{gathered} \tag{8}$$

Geometrically, the condition given in eq.(8) describes the intersection between the upper left triangle and the sigmoid curve in Supplementary Figure 3a. Depending on the parameter values of the sigmoid function (*i.e.* $\{\alpha, \rho, \eta\}$), this intersection either consists in a single region of the form $]-\infty, I_0]$ or in the union of two regions $]-\infty, I_0] \cup \mathcal{S}_s$, where $\mathcal{S}_s$ corresponds to the section of the sigmoid curve between the points I_1 and I_2, on Supplementary Figure 3b,c,d. This $\mathcal{S}_s$ region is of particular interest as it represents the admissible steady states describing seeds reaching a final size. We therefore investigated its existence and stability conditions.

2.2 Existence

Within the configuration space (r, k), the abscissae of the boundaries $\{I_0, I_1, I_2\}$ of the steady states region correspond to the the solutions of equation (9).

$$F(r) = 0 \tag{9}$$

The steady state existence conditions we seek can therefore be obtained through the study of these roots, noted $r_\infty^{(0)}, r_\infty^{(1)}$ & $r_\infty^{(2)}$ hereafter; and especially on their dependency on the parameter values $\{\alpha, \rho, \eta\}$.

2.2.1 Numerical estimations

Influence of the parameters $\{\alpha, \rho, \eta\}$. We first estimated numerically the solutions of eq.(9) for various values of the parameter set $\{\alpha, \rho, \eta\}$. For each of these three parameters, we swiped a range of values while keeping the two others at their reference values, see tab.(3).

[1]*i.e.* properties related to the *shape* of the equations rather than to a specific parametrization.

Results of this exploration are presented in Supplementary Figures 3e,f,g. This analysis has been performed within the jupyter notebook named `steady_state_analysis.ipynb`, available within the gitlab repository of the project, see the **Code availability** paragraph in section 3. This analysis revealed bounds for parameter values compatible with steady state existence since, from Supp. Fig. 3e,f,g we infer that paramters of the mechano-sensitive stiffening function should verify:

$$\begin{aligned} &\alpha \geq 4.8 \\ &\rho \in [2.1, 6.9] \\ &\eta \geq 2.9 \end{aligned} \tag{10}$$

Influence of the control variable p. One of the main results from our seed growth simulations is that a smaller pressure yields bigger seeds. We looked for a confirmation of this feature of our model by tracking the evolution of the steady state region as a function of the control variable p, Supplementary Figures 3h,i. We noticed that varrying the pressure value did not impact the existence of steady state solutions of our system but only their values. This conforted us in considering endosperm pressure as an external control variable rather than an internal parameter of our system.

2.2.2 Analytical study

Equation (9) cannot be solved analytically in a general manner. However, its piecewise linear approximation given in eq.(11) can.

$$\begin{gathered} \tilde{F}(r) = 0 \\ \text{with:} \\ \tilde{F}(r) = \begin{cases} 1 - rp & r \leq r_0 = \frac{\rho}{p}(1 - \frac{2}{\eta}) \\ 1 - rp + \frac{\alpha}{2}\big(1 + \frac{\eta}{2}(\frac{rp}{\rho} - 1)\big) & r \in [r_0, r_1] \\ 1 + \alpha - rp & r \geq r_1 = \frac{\rho}{p}(1 + \frac{2}{\eta}) \end{cases} \end{gathered} \tag{11}$$

As Supplementary Figures 3e,f,g suggest; roots of equations (9) and (11) – noted respectively $r_\infty^{(i)}$ and $\tilde{r}_\infty^{(i)},\ i \in [\![0, 2]\!]$ – follow the same trends. Furthermore, away from the bifurcation points[2] on Supp. Fig. 3e,f,g, they are close enough so that analytical expressions of the latters appear relevant and insightful to understand the properties of the formers. Such expressions are provided in equations (12).

$$\begin{cases} \tilde{r}_\infty^{(0)} &= \frac{1}{p} \\ \tilde{r}_\infty^{(1)} &= \frac{1}{p}\frac{\left(1 + \frac{\alpha}{2}(1 - \frac{\eta}{2})\right)}{(1 - \frac{\alpha\eta}{4\rho})} \\ \tilde{r}_\infty^{(2)} &= \frac{1+\alpha}{p} \end{cases} \tag{12}$$

[2]The place within the parameter space where the second and third roots ($I_1 \& I_2$) appear and spread apart.

From the expressions of $\tilde{r}_\infty^{(1)}$ & $\tilde{r}_\infty^{(2)}$ in eq.(12) one can extract the following existence constrain on the parameter set $\{\alpha, \rho, \eta\}$:

$$\begin{gathered} \tilde{r}_\infty^{(1)} \leq \tilde{r}_\infty^{(2)} \\ \Downarrow \\ \alpha \geq \rho\big(1 + \frac{2}{\eta}\big) - 1 \end{gathered} \tag{13}$$

Equation (13) defines a half-space of the parameter space of our dynamical system where steady state solutions exist.

2.3 Stability

One remaining question concerned the stability of the steady state region $\mathcal{S}_s$. A perturbative approach applied to eq.(7) in the vicinity of $\mathcal{S}_s$ yields the following linear system:

$$\begin{gathered} \partial_{\tilde{t}} \begin{bmatrix} \delta r \\ \delta k \end{bmatrix} = \boldsymbol{J} \cdot \begin{bmatrix} \delta r \\ \delta k \end{bmatrix} \\ \text{with:} \\ \boldsymbol{J} = \gamma \begin{bmatrix} 0 & 0 \\ \frac{\alpha\eta}{\rho} \frac{(r_\infty p/\rho)^{-\eta}}{\left(1+(r_\infty p/\rho)^{-(\eta+1)}\right)^2} & -1 \end{bmatrix} \end{gathered} \tag{14}$$

where $\boldsymbol{J}$ is the Jacobian of the system near the steady state. Its eigenvalues,

$$(\lambda_{\boldsymbol{J}}^{(0)}, \lambda_{\boldsymbol{J}}^{(1)}) = (0, -\gamma),$$

imply that $\mathcal{S}_s$ is indeed an attractor and the corresponding configurations are stable.

3 Numerical simulations

Together with a set of initial values $\{r_0, k_0\}$, equation (7) constitutes an *Initial Value Problem*. Given a set of values for the parameters $\{\alpha, \gamma, \rho, \eta\}$ and the control variable p, one can simulate the seed growth dynamics by resolving such an *IVP*.

To that end, we implemented system (7) in python (v 3.7.5) and make use of the `solve_ivp` method from the `scipy.integrate` module (v 1.3.1) to solve it given some initial conditions and pressure values. All simulations were performed between $t_{\text{min}} = 0$ and $t_{\text{max}} = 3$ with a time step $\delta t = 0.02$, where the temporal unit corresponds to the growth equation characteristic time (τ_{g}).

Initial state. We considered the following initial values for the dimensionless radius and effective stiffness of our model: $\{r_0, k_0\} = \{1.5, 1\}$. We chose $r_0 = 1.5$ based on inspection of experimental data. In their initial state, seeds display a very small endosperm and their radius is only slightly bigger than the thickness of their testa.

To set the condition $k_0 = 1$, we made the assumption that at the beginning of the growth process, the effective stiffness of the testa has not yet been subjected to any mechano-sensitive enhancement and was simply at its basal equilibrium value.

Matching simulation time with experimental time. The simulation time is measured in units of growth characteristic time (τ_{g}). This notion is rather arbitrary and does not need to be specified to perform simulations (only the ratio between growth and stiffening characteristic times, namely parameter γ is needed). However, in order to compare simulation results with experimental measurements, we set a *convertion factor* in order to express simulation times in *DPA*[3] unit:

$$\tau_{\text{g}} = 5\,\text{DPA} \tag{15}$$

Code availability: All simulation scripts as well as data analysis notebooks are freely available on line: `https://gitlab.inria.fr/mosaic/publications/seed_sup_mat`. A detailed description of how to install and run our simulations is provided within the `README.md` file within this repository. A visual representation of our digital workflow, from experimental data to figures files is provided as Supplementary Fig.15.

Data availability: Simulation scripts and notebooks require input data available on line as well: `https://zenodo.org/record/4620948#.YFR0Hi1h0UE`

[3] Days Post-Anthesis: a classic measure of seed development time.

3.1 Parameter space exploration

Rationale: As mentionned previously, we started this study with no clear assumptions concerning the values of the four parameters featured in the stiffening equation, second line of system (7).

We therefore investigated system (7) behavior for a wide range of parameter values, see table (4). Overall, we sampled our four dimensional parameter space into $5 \cdot 10^5$ parameter sets and simulated the dynamics of the system for all of them. All simulations, within this parameter space exploration, featured the same, constant value of relative pressure: $p = 1.2$, chosen arbitrary, slightly above the threshold value 1.

To that end, we used the python library `pypet` (*6*) to distribute and manage simulations over a multi-core computing server. Data management was performed using the `DataFrame` data structure from the `Pandas` library (v 0.25.3) (*7*). The raw results of this parameter space exploration are accessible within the zenodo repository associated with this manuscript (`/model/data/data_param_space_explo/`) and can also be re-generated by running the `explore_parameter_space.py` script within the `model/script/` directory in the gitlab repository. Data processing and analysis were performed within `Jupyter notebooks` (v 6.0.2) and visualization with the `seaborn` library (v 0.9.0) (*8*).

Selection of the parameter space region to investigate. Due to computational limitations, we had to limit the range of our parameter space exploration.

- the α parameter quantifies the amplitude of the *stress-sensitive* stiffening term, compared to the *passive* terms, in the second line of system (7). Theoretically, the only constrain on its value is that it belongs to $\mathbb{R}^+$. But AFM measurements performed during organogenesis at the Shoot Apical Meristem, reported 3 to 4 fold variations of wall stiffness value (*9*), suggesting that the stress-sensitive term should be significant but not overwhelming. To that end, we tested values between 1 and 11.

- We considered odd integer Hill function exponents (η) ranging from 3 to 9 to probe the influence of the non-linearity of the stress-sensitive stiffening term.

- The parameters γ and ρ correspond respectively to the ratios of the characteristic times and threholds between the growth and stiffening processes. We chose to sample them along a logarithmic scale, *i.e.* by setting $\gamma = 10^x$ and $\rho = 10^y$ and considering ranges centered on 0 for x and y. Precisely, we chose: $x, y \in [-2.5, 2.5]$. This enabled us to consider symmetric situations with respect to the kinetics of stiffening compared to growth.

For the three parameters α, ρ and γ we sampled the considered intervals into 50 points each. Combined with the four considered values for the parameter η, the four dimensional region we considered within the parameter space has been discretized into $5 \cdot 10^5$ samples, each corresponding to a unique set of values $\{\alpha, \eta, \rho, \gamma\}$.

This parameter space exploration is handled by the python script `explore_parameter_space.py`, available within the `model/script/` folder on the gitlab repository associated with this manuscript.

All the simulations performed during this parameter space exploration process are recorded in a `pypet` specific format within an `.hdf5` file entitled `raw_trajectory.hdf5` in a dedicated folder: `model/data/data_param_space_explo/YYMMDD_HHmmSS_seedgrowth/hdf5/` [4]. Note that the `/model/data/` folder does not initally exist in the repository and will be generated when the script is first launched.

Once the raw simulations have been performed, a second batch process is applied to them in order to format these results in the userfriendly data structure `Pandas.DataFrame`. This batch formatting process is handled by the `format_raw_result.py` script, also available in the `model/script/` directory of gitlab repository. The results of this formatting process are recorded within the same folder as the results of the parameter space exploration under the name `formatted_dataframe.hdf5`.

Result filtering. Once this systematic exploration done, we discarded simulations that did not meet the two following criteria:

- Simulations must have converged toward a steady value, first line of equation (16).
- The radius final value must lie within a range compatible with experimental measurements, second line of equation (16).

$$\begin{cases} \frac{\dot{r}}{r}|_{\infty} < 10^{-2} \\ \frac{r_{\infty}}{r_0} = 3.5 \pm 0.5 \end{cases} \tag{16}$$

Once this filtering done, less than $2 \cdot 10^3$ simulations remained.

This data triming process is performed in the `parameter_space_exploration.ipynb` notebook, located within the `/model/notebooks/` directory.

Comparison with experimental data. We then compared the relative radius dynamics of each kept simulation to experimental measurements. To that end, we first matched the simulation time with the experimental one by applying the following change of variable: $\tilde{t} \to t = \tau_{\mathrm{g}}\tilde{t}$ (with $\tilde{t}$ represents the simulation time), with the value for τ_{g} given in expression (15). Then, we sampled every simulations at integer time steps (corresponding to experimental sampling times): $t_k \in \{0, 1, 2, \dots 10\}$ and then measured the (root-mean-square) distance between the following vectors:

$$\begin{cases} \boldsymbol{s}_i = [\frac{r_i(t_1)}{r_i(t_0)}, \dots, \frac{r_i(t_{10})}{r_i(t_0)}]^t \\ \boldsymbol{e} = [\frac{R(t_1)}{R(t_0)}, \dots, \frac{R(t_{10})}{R(t_0)}]^t, \end{cases} \tag{17}$$

[4] `YYMMDD_HHmmSS` is a placeholder for a time-stamp generated each time the script is launched.

where the index i runs over all kept simulations and $R(t_k)$ depicts the mean value of the seed radii measured at time step t_k.

N.B.: *One can note that the simulation result vector* $\boldsymbol{s}_i$ *is constructed from the relative radius variable* $r = R/H$*, while the experimental measurement vector* $\mathbf{e}$ *is directly constructed from the seed radius estimation* R*. In order to compare them, we assumed seed coat thickness* (H) *constant during the seed expansion phase. This assumption was mainly motivated by the fact that the number of cell layers within the seed coat remains constant and that each cell layer, within the seed coat, roughly keeps the same thickness during the studied period.*

We then defined a fitting score ($F(i)$) for each simulation (i) as the inverse of the distance between the corresponding vector $\boldsymbol{s}_i$ and the vector of experimental measurements $\boldsymbol{e}$:

$$F \colon i \rightarrow F(i) = \frac{F_0}{||\boldsymbol{s}_i - \boldsymbol{e}||} \quad \text{with:} \quad ||\boldsymbol{v}|| = \sqrt{\sum_k v_k^2}, \tag{18}$$

where the constant $F_0 = \min(||\boldsymbol{s}_i - \boldsymbol{e}||)$ is used in order to normalize the fitting score to one for the best fitting simulation.

This fitting procedure enabled us:

- To visualize sub-regions of the parameter space corresponding to simulations matching the dynamical properties of actual seeds, see figure (1.D) within the main text and supplementary figure (S4).
- To sort all of the kept simulations by their degree of similarity to experimental data. And concentrate our analysis of parameter values on the hundred best-fitting simulations, see figures (1.B) and (3.A) within the main text.

We performed this fitting analysis against three sets of experimental measurements, corresponding to three different genotypes: wild type (ecotype Col-0), *iku2* and *ede1-3*. The results are given in table (5).

This comparison with experimental data and fitting analysis are also performed within the *parameter_space_exploration.ipynb* notebook, located within the `/model/notebooks/` directory.

The results, parameter values and various fitting scores are stored as a `Pandas.DataFrame` data structure within the following `.hdf5` file: `sim_res_XXX.hdf5` [5], located in the `/model/-data/results/` folder accessible on the gitlab repository associated with this manuscript.

3.2 Simulations with time dependent pressure

Rationale: While the assumption of constant pressure appears relevant at first, a closer look at experimental data suggested that endosperm pressure is a monotonously decreasing function of time, Fig.2d and Supplementary Fig.5b,c,d. Moreover, this *drop* of endosperm pressure over

[5] The placeholder *XXX* is set by the user.

time appeared more pronounced in WT seeds than in *iku2* seeds. Paradoxically, despite the shallower drop in endosperm pressure they experience; *iku2* seeds end up smaller than WT ones. Since *iku2* expression is restricted to the endosperm compartment, Fig.2a, this discrepancy between the WT and the *iku2* phenotypes should not directly result from altered seed coat properties. It cannot, therefore, be accounted for through different parameter values ($\{\alpha, \eta, \rho, \gamma\}$) within our model; as these parameters solely grasp properties of the seed coat. These experimental observations raised the following questions for our model:

- Can our model still produce realistic seed growth dynamics when fed with pressure-drop functions?
- How can the amplitude (*i.e.* depth) of this time-dependent drop impact the final size of the system?
- Could we mimic the phenotype discrepency between WT and *iku2*, within our model, using a common set of parameters but different pressure functions ?

***Modus Operandi*:** We developed a two-step approach: We first worked on a proof-of-concept by investigating the consequences of a generic time-dependent pressure drop compared to a constant pressure function. Then, we studied more realistic scenarios by considering time-dependent pressure functions extracted from experimental endosperm pressure measurements performed on both WT and *iku2* seeds.

Proof of concept: We first generated a reference set of simulations where pressure was assumed to be constant over time. Since *iku2* seeds displayed shallower pressure drop, Fig.2d and Supplementary Fig. 5b-d, we ran a parameter space exploration to fit constant-pressure simulations on *iku2* growth measurements. We kept the 100 simulations best-fitting the *iku2* growth data and considered the corresponding 100 sets of parameter values ($\{\alpha, \eta, \rho, \gamma\}$) as references.

Then, we ran simulations parametrized with these 100 reference sets but, this time, considereding a pressure function decreasing with time. In this first attempt, we considered a generic drop function only qualitatively comparable to experimental pressure measurements of WT seeds, eq.(19), blue graph in Supplementary Figure 5e, top panel. It consisted in a $\delta = 30\%$ drop from the initial value, spanned between 1 and 5 DAP ($\tau = 3\,DAP, n = 5$).

$$P_{\text{poc}}(t) = p_0 \left(1 - \frac{\delta}{1 + (t/\tau)^{-n}}\right) \tag{19}$$

Such a raw approach demonstrated that considering a time-dependent drop function as pressure input in our model yields bigger final radii than keeping the inital pressure value constant, Supplementary Fig.5e-f, bottom panel.

This first step of the analysis has been performed within the `influence_generic_pres--sure_drop.ipynb` notebook, located in the `/model/notebooks/` directory.

Realistic scenarios: To go further, we wondered if this result would hold if we considered realistic time-dependent pressure functions. To generate such functions, we first pooled together all the pressure measurements we had for both genotypes (WT and *iku2*) and we fitted them with piecewize linear functions, respectively noted $P_{\text{col}}(t)$ and $P_{\text{iku}}(t)$, Fig.2d and Supplementary Fig. 5b-d. To strengthen our study we complemented these two data-fitted pressure functions with three intermediate ones, directly interpolated from them, eq.(20), Fig.2e top panel.

$$P_{\nu}(t) = \nu P_{\text{col}}(t) + (1 - \nu) P_{\text{iku}}(t) \text{ with } \nu \in [0, 1]. \tag{20}$$

As before, we first generated a reference set of simulations by conducting a parameter space exploration. This time we used the *iku2* data-extracted interpolation function, $P_{\text{iku}}(t)$, as time-dependent endosperm pressure input. We kept the 100 simulations best-fitting the *iku2* growth data and considered the corresponding 100 sets of parameter values as references.

Then, we re-ran simulations on these 100 reference sets of parameter values but this time with the other pressure functions as inputs: $P_{\text{col}}(t)$ as well as $P_{\nu}(t)$ with $\nu \in \{0.25, 0.50, 0.75\}$. As the time-dependent pressure drop got deeper, we observed an increase of the seed final radii, in a range perfectly comparable to experimental measurements, Fig. 2e,f.

This final step of the time-dependent pressure analysis has been performed within the `influ--ence_realistic_pressure_drop.ipynb`, located in the `/model/notebooks` directory.

3.3 Modulation of the stiffening parameters

Rationale: The *iku2* phenotype can be rescued by inducing the mutation of *ap2*, a gene responsible of outer-integument differenciation, Fig. 4e, Supplementary Fig. 12c. Within our modeling approach, the influence of the *ap2* gene is accounted through modulation of the parameters of the mechano-sensitive stiffening function in eq. (7), either its strength (α), relative threshold (ρ) or Hill coefficient (η).

We consequently wandered how modulations of the stiffening parameters (α, ρ, η) would impact the final radius reached by our system.

Modus Operandi: As we did in the previous section when studying realistic scenarios, we used as reference the 100 simulations best-fitting *iku2* growth dynamics with $P_{\text{iku}}(t)$ pressure function as input.

From this reference, we envisonned two perturbative scenarios: First, while using the same pressure function $P_{\text{iku}}(t)$, we studied how the final states of these simulations were altered when the parameters of the stiffening function were modified. These simulations were ment to address the double mutant *iku2-ap2* case.

Secondly, we used the pressure function $P_{\text{col}}(t)$ pressure function as input; performed the modulations on the stiffening parameters and study how the simulations were altered. This time, the goal was to address the single mutant *ap2* case.

Simulating the *iku2-ap2* double mutants: Using the $P_{\text{iku}}(t)$ pressure function as input, we altered the stiffening parameters (α, ρ, η) as follow: for each reference set we modulated one by one the parameter values in a $\pm 20\%$ range and ran the corresponding simulations. For the sake of concision, we only kept four values of modulations: $-20\%, -10\%, 10\%, 20\%$,.

Simulating the *ap2* mutant: We re-ran the same simulation campaign but this time, using the $P_{\text{col}}(t)$ pressure function as input.

These two simulations campaigns have been performed within the `modulation_stif--fening_parameters.ipynb` notebook, located in the `/model/notebooks/` directory.

Simulations qualitatively agree with experiments: We reported how final radii, Fig. 4g, as well as growth dynamics, Supplementary Fig. 12d,e, were affected by stiffening parameter modulations. This simulation campaign showed that indeed, by modulating stiffening parameters around their reference values, we could generate stable growth dynamics leading to bigger or smaller final radii.

These results also highlighted that the final seed radius does not depend on all stiffening parameters with the same sensitivity. Indeed, modulations of the threshold ratio (ρ) yield the stronger variations while the same modulations of the Hill exponent (η) only slightly affected the final radius.

Simulations performed with $P_{\text{col}}(t)$ as input presssure function, yield slightly more altered final radii than the ones performed with $P_{\text{iku}}(t)$, this is mostly visible when the threshold ratio (ρ) is modulated, Fig. 4g.

Finally, as the diverging confidence intervals around some curves in Supplementary Fig. 12d,e suggest, in some cases, parameter modulation distabilized the simulation, leading to unrealistic dynamics. We tracked the number of these *outliers*, Supplementary Table 6, and limit the modulation amplitude to $\pm 20\%$ in order to keep it below 6% [6], *i.e.* at most 6 diverging simulations over the 100 simulations performed in total for each parameter modulation.

To sum up, by increasing the threhold ratio ρ by 20% in simulations mimicking *iku2* growth dynamics, we almost retrieved final radius values comparable to simulations mimicking WT behavior. A similar modulation performed on simulations mimicking WT behavior also yield bigger seeds, qualitatively agreeing with experimental observations of *ap2* seeds.

References

1. Lockhart, J. A. An analysis of irreversible plant cell elongation. *Journal of theoretical biology* **8**, 264–275 (1965).

[6]value obtained with $P_{\text{col}}(t)$ when no modulation is applied to the stiffening parameters.

2. Ortega, J. K. Augmented growth equation for cell wall expansion. *Plant Physiology* **79**, 318–320 (1985).

3. Bassel, G. W. *et al.* Mechanical constraints imposed by 3D cellular geometry and arrangement modulate growth patterns in the Arabidopsis embryo. *Proceedings of the National Academy of Sciences of the United States of America* **111**, 8685 – 8690 (2014).

4. Boudon, F. *et al.* A computational framework for 3D mechanical modeling of plant morphogenesis with cellular resolution. *PLoS Computational Biology* **11**, e1003950 (2015).

5. Zhao, F. *et al.* Microtubule-Mediated Wall Anisotropy Contributes to Leaf Blade Flattening. *Current Biology* (2020).

6. Meyer, R. & Obermayer, K. pypet: A Python Toolkit for Data Management of Parameter Explorations. *Frontiers in Neuroinformatics* **10**, 30–15 (2016).

7. Reback, J. *et al.* pandas-dev/pandas: v1.0.0rc0 (2020). URL `https://doi.org/10.5281/zenodo.3603473`.

8. Waskom, M. *et al.* mwaskom/seaborn: v0.9.0 (july 2018) (2018). URL `https://doi.org/10.5281/zenodo.1313201`.

9. Milani, P. *et al.* In vivo analysis of local wall stiffness at the shoot apical meristem in Arabidopsis using atomic force microscopy. *The Plant journal : for cell and molecular biology* **67**, 1116 – 1123 (2011).
