# Supplementary material for: Evidence that endosperm turgor pressure both promotes and restricts seed growth and size
Source: Nat Commun. 2023 Jan 5;14:67. doi: 10.1038/s41467-022-35542-5 (PMC9814827; doi:10.1038/s41467-022-35542-5)
Supplement: Supplementary file 3 — Reporting Summary [file 41467_2022_35542_MOESM3_ESM.pdf]

nature portfolio

Corresponding author(s): Olivier Ali, Gwyneth Ingram and Benoit Landrein

Last updated by author(s): 2022/12/05

Reporting Summary

Nature Portfolio wishes to improve the reproducibility of the work that we publish. This form provides structure for consistency and transparency in reporting. For further information on Nature Portfolio policies, see our Editorial Policies and the Editorial Policy Checklist.

Statistics

For all statistical analyses, confirm that the following items are present in the figure legend, table legend, main text, or Methods section.

n/a	Confirmed	
☐	☒	The exact sample size (*n*) for each experimental group/condition, given as a discrete number and unit of measurement
☐	☒	A statement on whether measurements were taken from distinct samples or whether the same sample was measured repeatedly
☐	☒	The statistical test(s) used AND whether they are one- or two-sided<br>*Only common tests should be described solely by name; describe more complex techniques in the Methods section.*
☐	☒	A description of all covariates tested
☒	☐	A description of any assumptions or corrections, such as tests of normality and adjustment for multiple comparisons
☐	☒	A full description of the statistical parameters including central tendency (e.g. means) or other basic estimates (e.g. regression coefficient) AND variation (e.g. standard deviation) or associated estimates of uncertainty (e.g. confidence intervals)
☒	☐	For null hypothesis testing, the test statistic (e.g. *F*, *t*, *r*) with confidence intervals, effect sizes, degrees of freedom and *P* value noted<br>*Give P values as exact values whenever suitable.*
☒	☐	For Bayesian analysis, information on the choice of priors and Markov chain Monte Carlo settings
☒	☐	For hierarchical and complex designs, identification of the appropriate level for tests and full reporting of outcomes
☒	☐	Estimates of effect sizes (e.g. Cohen's *d*, Pearson's *r*), indicating how they were calculated

Our web collection on statistics for biologists contains articles on many of the points above.

Software and code

Policy information about availability of computer code

Data collection	The experimental data generated for this article are available on Zenodo and can be found following this link: https://doi.org/10.5281/zenodo.7078846. They can also be found on the server of the laboratory of Plant Development and Reproduction (https://flower.ens-lyon.fr/). The experimental measurements used to generate the figures of this article are provided in the Source Data file.
Data analysis	All of the data were analyzed using Excel (v. 2016), the R software (v.4.2.0), or Python (3.7.5). All the code is available at https://gitlab.inria.fr/mosaic/publications/seed_sup_mat. Exact p-values are presented in the Source Data File (Statistical Analysis).

For manuscripts utilizing custom algorithms or software that are central to the research but not yet described in published literature, software must be made available to editors and reviewers. We strongly encourage code deposition in a community repository (e.g. GitHub). See the Nature Portfolio guidelines for submitting code & software for further information.

Data

Policy information about availability of data

All manuscripts must include a data availability statement. This statement should provide the following information, where applicable:

- Accession codes, unique identifiers, or web links for publicly available datasets
- A description of any restrictions on data availability
- For clinical datasets or third party data, please ensure that the statement adheres to our policy

All the code is available at https://gitlab.inria.fr/mosaic/publications/seed_sup_mat. All the experimental data is available in the main text, in the supplementary materials, source data file, or at: https://doi.org/10.5281/zenodo.7078846.

Human research participants

Policy information about studies involving human research participants and Sex and Gender in Research.

Reporting on sex and gender	NA
Population characteristics	NA
Recruitment	NA
Ethics oversight	NA

Note that full information on the approval of the study protocol must also be provided in the manuscript.

Field-specific reporting

Please select the one below that is the best fit for your research. If you are not sure, read the appropriate sections before making your selection.

[x] Life sciences   [ ] Behavioural & social sciences   [ ] Ecological, evolutionary & environmental sciences

For a reference copy of the document with all sections, see nature.com/documents/nr-reporting-summary-flat.pdf

Life sciences study design

All studies must disclose on these points even when the disclosure is negative.

Sample size	No sample size calculation was performed prior to the experiments. The number of seeds (biological replicate) analyzed in each experiment was chosen given technical constraints (in time and material) specific to each experiment.
Data exclusions	In the experiments involving imaging of seeds by optical and confocal microscopy, seeds that were strongly injured during sample preparation were excluded from the analysis. In the piercing experiments, only seeds presenting at least one wall pierced in the center were included.
Replication	Each experiment was carried independently twice at least (i.e. from independent batches of plants growing at different times). Data from independent experiments were pooled except in the following cases: 1. when the growth conditions were different, 2. when the settings used for the acquisition of fluorescent images were not the same, 3. where sample sizes were very different between independent experiments.
Randomization	No randomization was performed.
Blinding	No blinding was performed.

Reporting for specific materials, systems and methods

We require information from authors about some types of materials, experimental systems and methods used in many studies. Here, indicate whether each material, system or method listed is relevant to your study. If you are not sure if a list item applies to your research, read the appropriate section before selecting a response.

Materials & experimental systems

n/a	Involved in the study
☐	☒ Antibodies
☒	☐ Eukaryotic cell lines
☒	☐ Palaeontology and archaeology
☒	☐ Animals and other organisms
☒	☐ Clinical data
☒	☐ Dual use research of concern

Methods

n/a	Involved in the study
☒	☐ ChIP-seq
☒	☐ Flow cytometry
☒	☐ MRI-based neuroimaging

Antibodies

Antibodies used	LM19 primary antibody (monoclonal rat antibody, AB_2734788, PlantProbes) revealled with secondary antibody (anti-rat IgM mu Chain Dylight 488, AB_1660991 (ab96693), Abcam). JIM5 primary antibody (monoclonal rat antibody, PlantProbes) revealled with secondary antibody (anti-rat IgG Alexa Fluor 488, AB_2535796 (A21210), Thermo Fischer Scientific). 2F4 primary antibody (mouse primary antibody, PlantProbes) revealled with secondary antibody (anti-mouse IgG Alexa Fluor 488, AB_141607 (A21202), Molecular Probes)
Validation	LM19: Verhertbruggen et al. (2009) Carbohydr. Res. 344, 1858; JIM5: VandenBosch et al. (1989) EMBO Journal 8, 335-342, Knox et al. (1990) Planta 181, 512-521, Willats et al. (2000) Carbohydr. Res. 327, 309-320, Clausen et al. (2003) Carbohydr. Res. 338, 1797-1800; 2F4: Liners et al. (1989) Plant Physiology 91, 1419-1424.
